# Multispecies autocatalytic RNA reaction networks in coacervates

**DOI:** 10.1038/s42004-023-00887-5

**Published:** 2023-05-08

**Authors:** Sandeep Ameta, Manoj Kumar, Nayan Chakraborty, Yoshiya J. Matsubara, Prashanth S, Dhanush Gandavadi, Shashi Thutupalli

**Affiliations:** 1 Simons Centre for the Study of Living Machines, National Centre for Biological Sciences, Tata Institute of Fundamental Research, Bengaluru, Karnataka India; 2 International Centre for Theoretical Sciences, Tata Institute of Fundamental Research, Bengaluru, Karnataka India; 3 Present Address: Trivedi School of Biosciences, Ashoka University, Plot No. 2, Rajiv Gandhi Education City, P.O. Rai, Sonepat, Haryana 131029 India

**Keywords:** RNA, Origin of life

## Abstract

Robust localization of self-reproducing autocatalytic chemistries is a key step in the realization of heritable and evolvable chemical systems. While autocatalytic chemical reaction networks already possess attributes such as heritable self-reproduction and evolvability, localizing functional multispecies networks within complex primitive phases, such as coacervates, has remained unexplored. Here, we show the self-reproduction of the *Azoarcus* ribozyme system within charge-rich coacervates where catalytic ribozymes are produced by the autocatalytic assembly of constituent smaller RNA fragments. We systematically demonstrate the catalytic assembly of active ribozymes within phase-separated coacervates—both in micron-sized droplets as well as in a coalesced macrophase, underscoring the facility of the complex, charge-rich phase to support these reactions in multiple configurations. By constructing multispecies reaction networks, we show that these newly assembled molecules are active, participating both in self- and cross-catalysis within the coacervates. Finally, due to differential molecular transport, these phase-separated compartments endow robustness to the composition of the collectively autocatalytic networks against external perturbations. Altogether, our results establish the formation of multispecies self-reproducing reaction networks in phase-separated compartments which in turn render transient robustness to the network composition.

## Abstract (web summary)

Self-reproducing autocatalytic chemical reaction networks have the potential to demonstrate heritability and evolvability, however, achieving functional catalytic assembly and reaction networks within a phase-separated environment remains challenging. Here, the authors report multispecies chemical reaction networks of self-producing RNAs within charge-rich coacervates.

## Introduction

Compartments based on complex coacervates serve as an excellent model for protocells^1,2^ and have extensively been used to study a variety of chemical reactions^3,4^. Under out-of-equilibrium conditions that allow for constant exchange of material with the external environment while maintaining a core of encapsulated chemicals, coacervates could also propagate i.e. grow and divide^4–7^. For such a compartmentalized physico-chemical system to exhibit life-like properties, it must enclose a robust, heritable identity that is propagated by growth and division, allowing it to participate in Darwinian-like chemical evolution^8–14^. However, endowing coacervate compartments with robust and heritable identities with the potential for evolution is not trivial. Broadly, such a compartmental identity can be achieved in two ways: (i) using a template-based replicating system, e.g., replicase ribozyme^15,16^ or (ii) by the chemical composition of self-reproducing chemical networks^17^. For the latter, also known as autocatalytic reaction networks (ACSs)^18,19^, the chemical species (often catalytic) collectively reproduce by synthesizing each other from a pool of substrates. In such networks, the steady-state composition (relative proportion of each species) serves as a chemical identity of the network (i.e. network composition)^11,12,17,20,21^.

Achieving functional catalytic assembly and reaction networks within the condensed environment of phase-separated droplets is a challenging task^22^. It must be noted however that multiple catalytic activities such as RNA cleavage and template-based polymerization have been shown in these droplets^23–25^. Furthermore, recently, several studies have reported critical catalytic steps towards the construction of an evolvable coacervate-based protocell^4^, e.g., catalytic assembly of longer RNA in condensates^26^, RNA reproduction via ligase activity^27^. However, from these demonstrations alone, it is not clear whether multispecies chemical reaction networks of self-reproducing RNAs can be supported within the condensed, charge-rich environment of complex coacervates. Even the relatively simpler step, namely the (auto-)catalytic assembly of longer (larger) catalytic RNA from constituent fragments remains unexplored in the condensed environment of phase-separated coacervates. Localizing ACS chemistries into dynamic coacervate compartments can open up the possibilities of a life-like evolvable chemical system—while the compartment protects the enclosed chemistry, a dynamic flux across the compartment boundary can sustain a balance between the ACS network robustness and possibility for the change (i.e. evolvability).

**Fig. 1:**
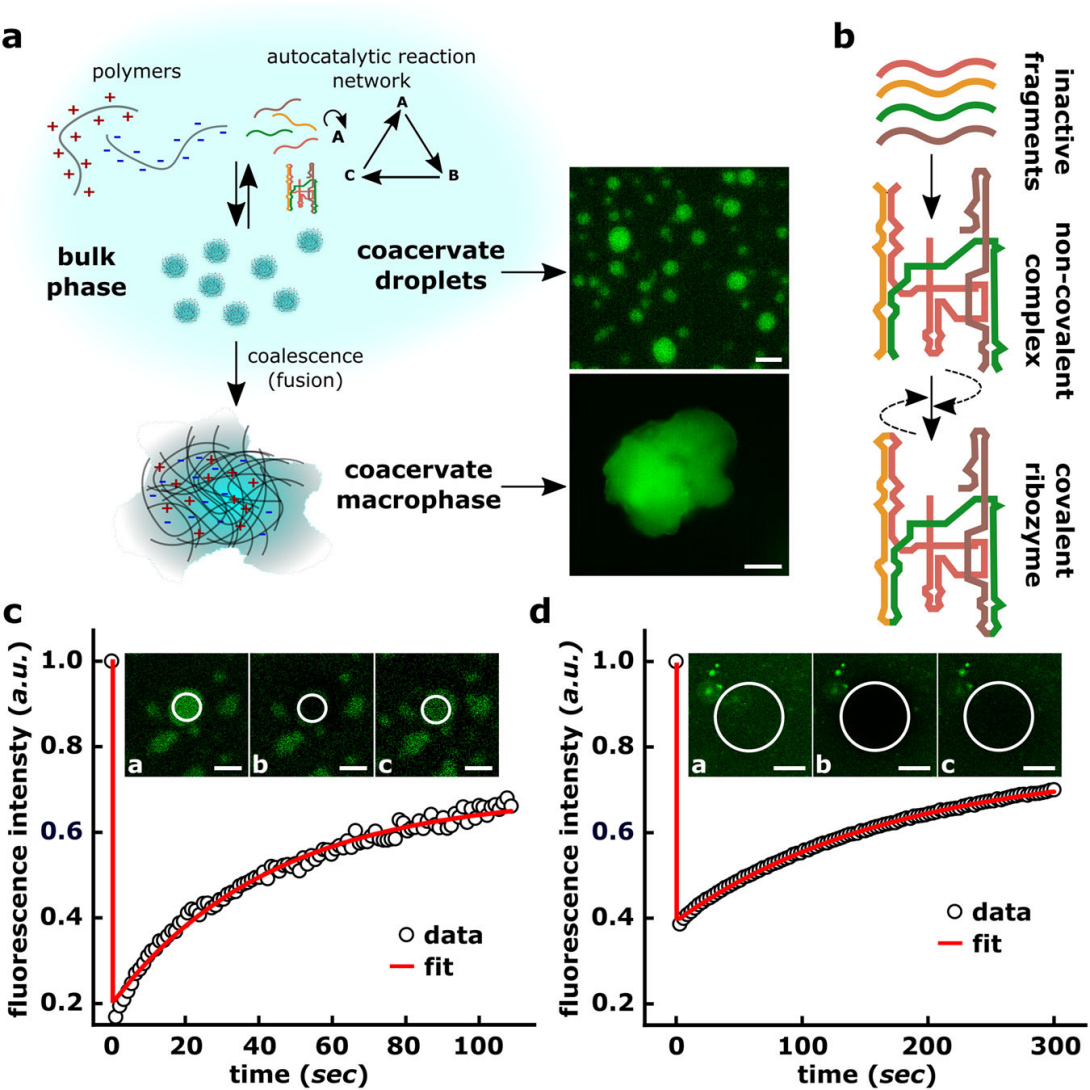
Conceptual schematic of our work involving coacervate compartments and the *Azoarcus* ribozyme system. **a** Schematic representation of phase-separated compartments formed by interactions between oppositely charged polymers (PAA and spermine, see “Methods”). These phase-separated compartments can exist as spatially isolated, micron-sized “coacervate droplets” in solution or can coalesce into a single consolidated “coacervate macrophase”. Both these coacervate environments support autocatalytic RNA self-assembly and reaction network formation. Microscopy images (green fluorescence channel) of PAA-spermine coacervate droplets (top right, scale bar 5 μm) and coacervate macrophase (bottom right, scale bar 10 μm). The fluorescence is due to 30-nt long oligonucleotides labeled with Alexa-488 added during the coacervation step (see “Methods”, Supplementary Table S1). **b** Schematic showing the self-assembly of *Azoarcus* covalent ribozyme (WXYZ, ~200 nt) from its inactive substrate RNA fragments (red: W ~ 65 nt, yellow: X ~ 43 nt, *green*: Y ~ 52 nt, brown: Z ~ 55 nt)^33,36^. The 5'-end of W and 3'-end of W, X, and Y contain 3-nt long recognition elements annotated as “IGS” and “tag'', respectively. When mixed together, the substrate RNA fragments rapidly self-assemble to form a non-covalent complex (catalytically active) which is converted to a covalent ribozyme (catalytically active, WXYZ). The dashed arrow denotes catalytic feedback from the non-covalent as well as covalent catalysts. **c** FRAP (fluorescence recovery after photobleaching) is used to characterize the diffusive dynamics in coacervate droplets **c** and in coacervate macrophase **d**. For both, Alexa-488-labeled 30-nt long oligonucleotide is used and experiments are performed at 48 °C. Insets show the sample images at pre-bleaching (*a*), bleached (*b*), and post-recovery (*c*) step of the FRAP experiment, scale bar 5 μm. See “Methods” for details.

We use fragments of the *Azoarcus* group I intron ribozyme^28^ to demonstrate autocatalytic assembly of longer catalytic RNA molecules within complex, charge-rich coacervate phases (Fig. 1). While phase-separated coacervates have typically been thought of as spherical micron-sized, liquid-like droplets, chemical dynamics in coacervates can occur in multiple settings^29^—in addition to droplets, coacervates can also be gel-like irregular particles, or even condensed bulk phases resulting from the coalescence of these structures^29^ (Fig. 1a). We therefore establish autocatalytic self-assembly and ACSs both in spatially isolated droplets and a coalesced coacervate ‘macrophase’ (Fig. 1a, b). Briefly, in this paper, (i) we demonstrate the self-assembly of large RNA catalytic molecules from constituent smaller fragments inside charge-rich coacervates (ii) this assembly can be achieved in both self-autocatalytic and collective cross-catalytic fashion, allowing us to form ACSs within coacervates, (iii) these ACS confer a chemical compositional identity to the coacervate compartments, and (iv) despite the lack of an impermeable boundary, the compartments transiently protect the enclosed reaction networks (and by extension, the chemical identity) from external perturbation. Altogether, by combining RNA autocatalytic networks with dynamic phase-separated coacervate compartments, our work opens the possibilities for creating primitive chemical units, that can participate in self-reproduction, growth, and division^6,7,29^, and adaptive evolution^30^.

## Results

The *Azoarcus* ribozyme, WXYZ, is an ~200 nucleotide (nt) RNA (Fig. 1b) which can be split into inactive RNA fragments of varying lengths (two fragments^30–32^, four fragments^33^, five fragments^34^). These fragments can covalently assemble in full-length active ribozymes in the presence of Mg^2+^ ions using both catabolic^35^ and anabolic^36^ approaches via recombination reactions^33^. The assembly occurs in an autocatalytic manner i.e. the self-assembled ribozymes catalyze the subsequent assembly of fragments into other functional ribozymes to form autocatalytic networks capable of collective reproduction^36,37^. Further, the assembly is guided by specific base-pair interactions between a 3-nt long internal guide sequence (IGS, “GUG” in wild-type group I intron) at the 5’-end of the ribozyme with the complementary 3-nt stretch (tag “CAU”) at the 3’-end of the substrate RNA fragments. Variations in the IGS and tag sequences allow for the creation of a diversity of ribozymes, differ in their recognition element (IGS and tag) and catalytic efficiency^31^, that can be used to build cross-catalytic reaction networks^30,37^.

We prepared coacervates using a negatively charged long chain poly-acrylic acid (PAA) and positively-charged spermine creating liquid-like droplets of varying size (0.5–5 μm) (Fig. 1a, “Methods”, and Supplementary Fig. S1). The coacervate phase is a charge-rich environment, with complex rheological properties that can not only affect the mobility of molecules into (and within) the condensed phase but also their reaction dynamics. Therefore, at first, by using fluorescently labeled oligonucleotides (Fig. 1a, right), we confirmed that negatively charged polymers (such as RNA or DNA) can spontaneously partition into the coacervate phase. As mentioned earlier, coacervates broadly include both micron-sized liquid-like droplets as well as phases that result from the coalescence of these droplets which is typical in many physical settings^29^. Specifically, we refer to a surface-attached coalesced bulk phase as a “coacervate macrophase”. First, we characterize the dynamics within both these coacervate phases using FRAP (fluorescence recovery after photobleaching) measurements. Our measurements confirm the diffusive dynamics of the oligonucleotides and dynamic exchange with the external environment in both phases (*droplet*: recovery time *τ* = 21.3 ± 3.0 s; diffusivity *D* = 0.1 ± 0.02 μm^2 ^ s^−1^, *macrophase*: recovery time *τ* = 172.5 ± 4.0 s and diffusivity *D* = 98 ± 1.8 × 10^−3^ μm^2 ^ s^−1^, Fig. 1c, d; “Methods”). These measurements clearly show that the droplet environment is more diffusive than the macrophase.

**Fig. 2:**
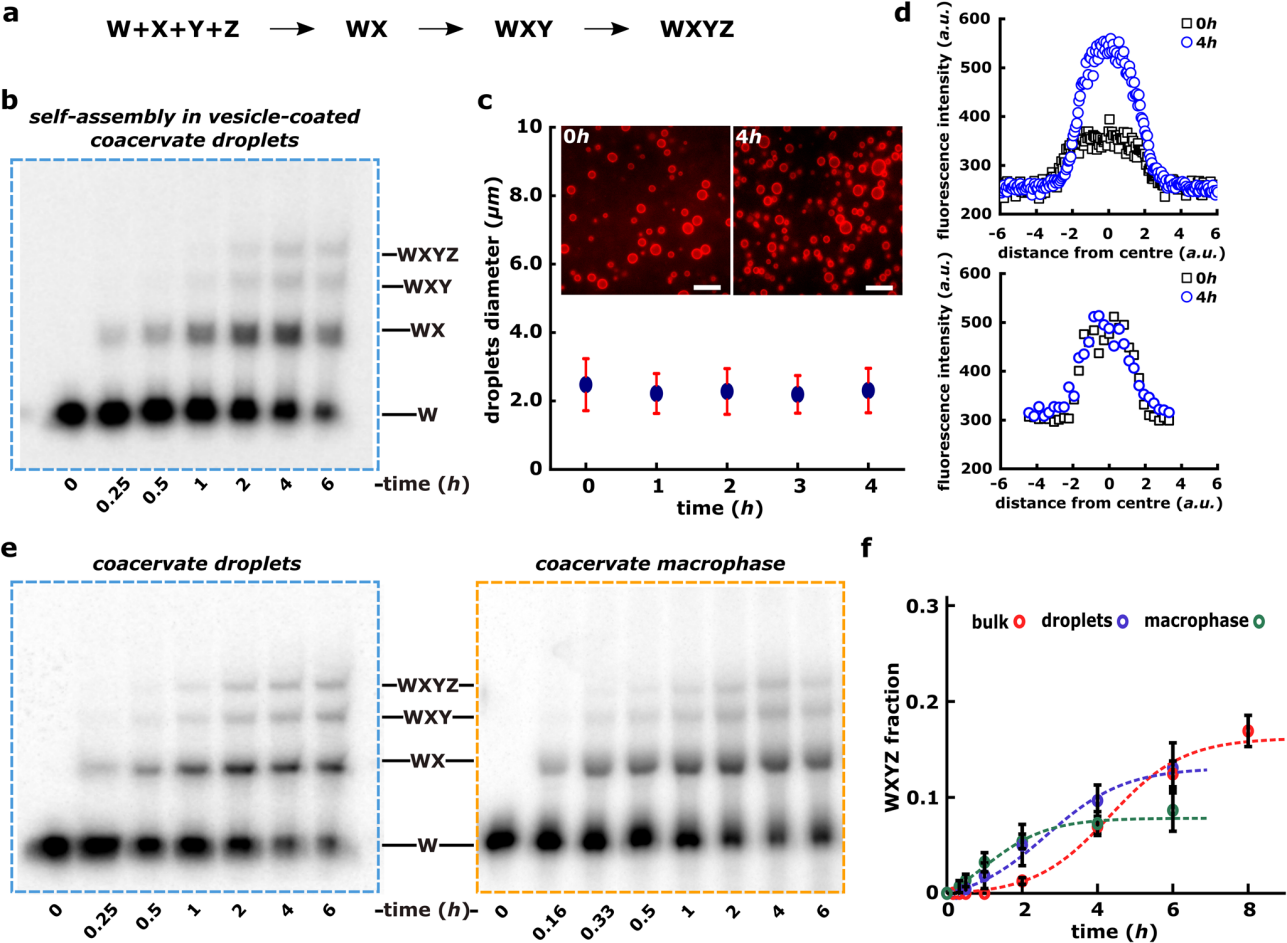
Autocatalytic self-assembly of the *Azoarcus* ribozyme in coacervates. **a** Schematic showing the systematic assembly of covalent ribozyme (WXYZ) from smaller inactive RNA fragments W, X, Y, Z via WX, WXY intermediates. **b** Polyacrylamide gel showing the self-assembly of *Azoarcus* ribozyme (WXYZ) from its small RNA fragments (W, X, Y, Z) inside the vesicle-coated coacervate droplets demonstrating that WXYZ formation indeed occurs inside the micron-size droplets. Here coacervate droplets were prepared together with RNA fragments (W, X, Y, and Z) and then coated with lipid vesicles (DOPC) prior to incubating at 48 °C and analyzed over polyacrylamide gel (see “Methods”). **c** Graph showing the stability of vesicle-coated coacervates during the reaction time course shown in **b**. Here average droplet diameter is plotted over the time. The microscopy images of vesicle-coated coacervate droplets (at 0 h and 4 h) are shown in inset. The fluorescence is due to the doping of 1,2-dioleoyl-sn-glycero-3-phosphoethanolamine-N-(lissamine rhodamine B sulfonyl) with the DOPC lipid (see “Methods”). **d** Graph showing the leakiness of the vesicle-coated coacervate used for the self-assembly reaction shown in **b**. The leakiness is tested as increase in fluorescence intensity of the coacervate droplets due to the diffusion of a 30-nt Alexa-488 DNA oligonucleotide (same as used in FRAP studies above, Fig. 1) from bulk solution to the vesicle-coacervate droplets. Leakiness is measured either for the 30-nt Alexa-488 DNA oligonucleotide alone (top) or hybridized to the ~200-nt *Azoarcus* ribozyme (*bottom*). Here the fluorescence intensity is plotted against distance from the center of the droplet. See “Methods” for the details. **e** Polyacrylamide gels showing the time course for the formation of full-length product (covalent ribozyme, WXYZ) starting from smaller RNA fragments inside coacervate droplets (left) as well as inside coacervate macrophase (right). The reaction samples are doped with (*γ*^32^*P*) labeled W RNA fragment (0.01 μM) added and folded together with all the fragments (W, X, Y, and Z, 0.75 μM each). See “Methods” for the experimental details. After incubation, samples are processed and analyzed via 12% denaturing PAGE. **f** Time courses showing the autocatalytic self-assembly of WXYZ ribozyme from W, X, Y, and Z RNA fragments in coacervate macrophase (green circles), coacervate droplets (blue circles) and as well as in bulk aqueous phase control (red circles). Reactions are done by encapsulating the substrate fragments in coacervates, separating the macrophase, or by incubating the droplets directly at 48 °C (Supplementary Fig. S2 and “Methods”). All the time courses are measured in triplicates and mean WXYZ product formation is plotted along with standard deviation. The time-course data is also analyzed by a kinetic model described for the *Azoarcus* assembly earlier^36^ and the fitted curves are shown as dotted lines (green, blue, and red for the macrophase, droplets, and bulk aqueous phase, respectively).

The partitioning of negatively charged oligonucleotides into the coacervates does not however guarantee their functionality, i.e., catalytic activity in the charge-rich and confining environment of coacervate droplets as well as that of the coalesced macrophase^29^. Identifying and quantifying any ribozyme activity inside such a charge-rich environment necessitates the careful distinction between reactions occurring inside the coacervate droplets, macrophase, and in the bulk solution phase (Fig. 1a). We, therefore, developed a protocol to separate the bulk of the solution from the coacervate phase and systematically analyzed the ribozyme activity within droplets as well as macrophase (“Methods” and Supplementary Fig. S2). Briefly, coacervates droplets were prepared encapsulating all the four RNA fragments (W, X, Y, and Z; Fig. 2a) and incubated at 48 °C. Indeed droplets are stable at such higher temperatures of the reaction though the number density decreased notably (Supplementary Fig. S3). To measure activity from coacervate droplets, the bulk aqueous phase was removed using brief centrifugation after the incubation (“Methods”; Supplementary Fig. S2) and separated phase was analyzed using polyacrylamide gels (“Methods”). Similarly for the coacervate macrophase, the bulk aqueous phase was separated prior to the incubation at 48 °C and only the separated coacervate macrophase (with no bulk solution around) was incubated ensuring RNA catalysis inside the coacervate macrophase.

First, in order to confirm that the ribozyme assembly can occur inside micron-sized coacervate droplets and to rule out the diffusion of the assembled product from bulk aqueous solution present around into the droplet, we devised an experiment in which the coacervate droplets containing RNA fragments were coated with lipid vesicles. The lipid vesicles coating of droplets restrict the RNA molecular transport from the bulk to the droplets (“Methods”). Therefore, the formation of full-length WXYZ within these vesicle-coated coacervate droplets unambiguously confirms that the reactions occur inside the micron-sized coacervate droplets (Fig. 2a, b). In addition, we have verified that these vesicle-coated coacervate droplets are stable against coalescence (Fig. 2c) and that the longer WXYZ RNAs cannot partition from the bulk (Fig. 2d) at 48 °C for longer duration. Altogether, these results affirm that the coacervate phase, even in the form of micron-sized droplets, can sustain the RNA self-assembly reactions.

Next, we systematically analyzed the assembly of WXYZ ribozyme inside membraneless coacervate droplets (i.e. without any vesicle coating) as well as inside the macrophase. Following the same protocol mentioned above, in both the cases, appearance of slower-moving products on a polyacrylamide gel corresponding to the size of full-length WXYZ, confirms the assembly of longer RNAs (*Azoarcus* ribozyme, Fig. 2e). The autocatalytic nature of the fragment assembly is evident in all cases: bulk solution phase, coacervate droplets as well as coacervate macrophase, as the time course of the WXYZ product formation follows a sigmoidal trend that is expected for autocatalytic growth in a closed system^33^ (Fig. 2f, red, blue and green data-points, see Supplementary Note 1). In addition, we have also analyzed the four-fragment assembly of WXYZ in the presence of the product of the reaction (full-length covalent WXYZ) seeded at the start of the reaction confirming the positive feedback in the assembly (Supplementary Fig. S4).

In order to explain the kinetic behavior of the assembly, we used a kinetic model, simplified from one described for the *Azoarcus* assembly previously^36^, to analyze the time-course data (Fig. 2f, red, blue and green dotted lines). Briefly, we considered the instantaneous assembly of a non-covalent complex from the four fragments (W, X, Y, and Z)^30,37^, and a (reversible) conversion reaction of W:X:Y:Z into a covalent catalyst (WXYZ) catalyzed by both WXYZ and W:X:Y:Z with different catalytic efficiencies, *k*_*a*_ and *k*_*b*_, respectively. In the chemical reaction model, which is reduced from the full detailed model described in refs. ^33,36^, we assumed two types of reactions:

1. A instantaneous non-covalent complex (W:X:Y:Z) formation from the four fragments (W, X, Y, and Z),
  $${{{{{{{\rm{W}}}}}}}}+{{{{{{{\rm{X}}}}}}}}+{{{{{{{\rm{Y}}}}}}}}+{{{{{{{\rm{Z}}}}}}}}\to \,{{\mbox{W:X:Y:Z}}}\,,$$
  whose association rate (estimated as ~10^2^–10^3^ min^−1^ for 1 μM of fragments, in the previous studies under similar condition^35–37^) is much faster than the timescale of the measurements. While its dissociation rate is much slower (estimated as ~10^−2^ min^−1^(see ref. ^37^)), thus the fraction of the fragments forming the complex is saturated, since the dissociation constant is far smaller than the concentration of the fragments ~1 μM.
2. Reversible covalent formation,
  $$\,{{\mbox{W:X:Y:Z}}}\rightleftharpoons {{\mbox{WXYZ}}}\,,$$
  is catalyzed both by W:X:Y:Z and WXYZ with rates *k*_*a*_ and *k*_*b*_, respectively. The ratio between the forward and the backward reactions is 1: *δ*.

Here, we also assume the four fragments (W, X, Y, and Z) and the ribozyme (WXYZ) do not decay during the observation. We set the equal molar of fragments as the initial condition. Then, defining the initial fraction of each fragment as unity, the sum of the fraction of W:X:Y:Z and WXYZ is conserved as unity since the complex formation is immediate, and all of the fragments exist as either W:X:Y:Z or WXYZ.

Under the above assumptions, the model rate equation is given as:

$$\frac{dx}{dt}=((1-x)-\delta x)({k}_{a}x+{k}_{b}(1-x)),$$

where 1 − *x* and *x* are the fraction of W:X:Y:Z and WXYZ, respectively. This can be simply transformed into the logistic differential equation,

$$\frac{dx}{dt}=(c-x)(ax+b),$$

where *c* = 1/(1 + *δ*), *a* = (1 + *δ*)(*k*_*a*_ − *k*_*b*_), and *b* = (1 + *δ*)*k*_*b*_. The solution for *x* is given as

$$x(t)=\frac{\exp ((a+bc)t)-1}{\exp ((a+bc)t)/c+a/b}.$$

The time course data obtained by the gel electrophoresis data (Fig. 2f) is fit to the above solution by minimizing the sum of weighted squared residuals^38^. Note that, as a result of the fitting, *a* > 0 (i.e., *k*_*a*_ > *k*_*b*_) indicates the evidence of autocatalytic reaction (Supplementary Table S2), i.e., the product ribozyme formation as a result of positive feedback from the product itself.

The model parametrizes only three quantities: the strength of positive feedback due to the formation of a catalytic product *a*, the rate of the background reaction (catalyzed by the non-covalent complexes) *b*, and the product fraction at equilibrium *c* (Supplementary Table S2). The data from all the different experiments fit equally well using this model and a sigmoidal nature of the time course is evident (Fig. 2f and Supplementary Fig. S5), thus demonstrating the autocatalytic nature of the RNA ribozyme assembly. In addition to this kinetic modeling of the time course data, by measuring the initial reaction rates of the WXYZ assembly from two fragments (WXY and Z) seeded with different initial concentrations of WXYZ as described earlier^30,31^, we confirmed positive feedback from the product of the reaction (WXYZ), further verifying the autocatalytic nature of the WXYZ assembly (Supplementary Fig. S6 and Supplementary Note 1, “Methods”). In addition, we also observed rate enhancement of *Azoarcus* ribozyme assembly inside the coacervate compartments compared to bulk aqueous control (Fig. 2f, Supplementary Note 2, Supplementary Figs. S7 and S8).

Altogether, these results show that coacervate compartments support the autocatalytic assembly of catalytic RNAs from smaller RNA fragments despite the condensed and charge-rich conditions—it must be emphasized here that this assembly not only requires the coming together of the various fragments but also the final assembled structure to adopt a functional secondary structure for further catalytic action.

We next turn our attention to the formation of cross-catalytic networks of ribozymes inside the coacervate compartments. Cross-catalysis, as discussed earlier, is important for the construction of larger and more diverse autocatalytic reaction networks with properties crucial for network robustness, self-reproduction, and Darwinian-like evolution of autocatalytic chemical systems^11,12,20,30,39^. The most striking manifestation of cross-catalysis involves a poor self-assembling species, the formation of which can be catalyzed by other chemical species—the growth of such a species is significantly enhanced due to cross-catalysis and is slow otherwise.

**Fig. 3:**
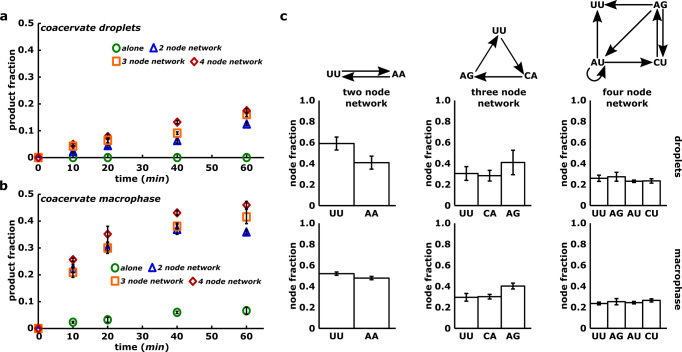
Formation of cross-catalytic networks inside coacervates. **a** Time courses showing the assembly of node UU as single node (alone, green circle), connected in a two nodes (blue triangle), three nodes (orange square) and four nodes network (red rhombus) in coacervate droplets. Network structures are shown in **c** (top). **b** Same as **a** but in the coacervate macrophase. All the time courses are measured in triplicates and mean WXYZ product formation is plotted along with standard deviation. **c** Bar-graphs showing the composition of two (left column), three (middle column), and four nodes network (right column). Network structures are shown at the top of each column. In these networks, arrow represents a directed edge showing the catalysis of a downstream node (WXYZ catalyst) by upstream node(s) (WXYZ catalyst) from its respective substrate fragments^30,32,37^. Here, compositions are represented as relative fraction of each network species (node) in the network and measured by quantifying the amount of each species formed in the network. All measurements were performed in triplicates and mean WXYZ product formation of each is plotted along with standard deviation.

To test this, we chose a poor self-assembler (IGS-tag combination of GUG and CUU respectively; denoted as UU species^31^) compared, for example, with another good self-assembling species (e.g., UA; Supplementary Fig. S9). We then designed cross-catalytic networks of different architectures and sizes (containing either two, three or four nodes) in such a way that the synthesis of the poor self-assembler UU is dependent on the catalysis by other members of the network (Fig. 3). For example, in the case of two nodes network, both UU and AA are poor self-assembler but can cross-catalyze each other by U → A link and A → U link, respectively. To this end, all the RNA fragments required for a particular catalytic reaction network are encapsulated in the coacervate and analyzed in droplets as well as macrophase. A radioactive labeled WXY fragment for the formation of the UU species is used to monitor the growth of the node corresponding to the poor self-assembling species in each of the networks. The time courses, denoting the assembly of the UU species, clearly indicate significant enhancement in both coacervate droplets and macrophase (Fig. 3a, b). In the case of coacervate macrophase up to 50% of the substrate converted into the product when UU is connected in network compared to 5% when UU is alone (Fig. 3b). These results show the assembly when the poorly self-assembling species is part of a cross-catalytic network; this is a clear demonstration of cross-catalytic reaction networks within the coacervate droplet and macrophase. Further, the production of the UU species due to the catalysis of two catalytic links (for e.g., in a four-node network) is enhanced when compared to production due to one catalytic link alone (for e.g., in two-, three-node networks); this is further confirmation of cross-catalytic network formation (Fig. 3a, b, compare blue with orange and red curves).

In order to identify the chemical composition of each network, we further measured relative fraction of individual species of the two-, three- and four-node reaction networks (Fig. 3c) by encapsulating all the substrate fragments required for a network and quantifying the formation of all the nodes individually (“Methods”). The measurements reveal that the network composition are maintained within the coacervate droplets and as well as in coacervate macrophase, despite the charge-rich environment, and the increased RNA concentration within the compartment (Fig. 3c). The relative proportions of the nodes in networks are indeed well correlated with those measured in the aqueous phase alone (Supplementary Fig. S10). Slight differences in some of the compositions may be due to the concentration dependant non-linearities of the reaction network kinetics and the transport within the condensed compartments.

Compartmentalization can be crucial for the subtle balance between the protection of the chemical properties of the compartment and the external chemical perturbations that can drive chemical evolution. Such a balance between robustness of the compositional identity and ability to explore new chemical states (that allows for the networks to expand by the addition of new species/nodes) have been shown to be crucial for Darwinian evolution to occur in the chemical reaction networks^30^. Such a scenario can also be tested using phase-separated compartments since they can be maintained as individual entities and as well as, are amenable to dynamic fusion-fission events^29^. However, we wondered whether membraneless compartment system such as coacervates can impede the perturbation to the chemical composition of autocatalytic reaction networks.

**Fig. 4:**
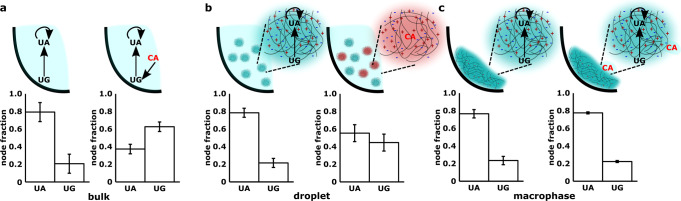
Robustness of cross-catalytic RNA networks in coacervates. Compositions are measured for a two-node cross-catalytic network in the absence or presence of a perturbing species under three different conditions. In this network, UA is self-catalyzed, as well as catalyzed by UG (via U → A link, **a**, top left). UG is also poorly self-catalyzed via U → G link (not shown). The perturbation is caused by adding a strong node (CA, shown in red) which catalyzes the synthesis of UG node (via C → G link, **a**, top right. **a** *Bulk*: Network is formed in the absence of any polymer and compositions are measured without perturbation (bottom left) as well as with perturbation (CA, bottom right). **b** Same as **a** but in coacervate droplets. Here, perturbing species (CA) is also encapsulated inside the coacervate droplets followed by mixing with droplets carrying the two-node network in 1:1 ratio, left without perturbation, right with perturbation. **c** Same as **b** but in coacervate macrophase. The perturbation is carried out by adding 0.5 μM solution of CA on top of macrophase, left without perturbation, right with perturbation. For each condition, samples were incubated for 30 min and then analyzed via polyacrylamide gel electrophoresis to measure the network compositions with or without perturbation. All the measurements are done in triplicates and mean WXYZ product formation is plotted as fraction w.r.t. each node along with standard deviation.

To test this, we designed a two-node reaction network containing the UA and UG ribozyme species (Supplementary Note 3). The UA and UG WXYZ ribozymes are connected by single catalytic edge from UG to UA via the U → A link^31^. WXYZ UG ribozyme catalyses the synthesis of WXYZ UA which in addition, is catalyzed by a self-loop (U → A link, Fig. 4a, top left). UG is also self-catalyzed by a weak wobble base-pair link (U → G link, not shown here). As expected, when network species were measured in the bulk aqueous phase, this arrangement resulted in a composition with a higher proportion of the UA species compared to UG species (Fig. 4a, bottom left). We then “perturbed” the network by the addition of a catalytic species CA that catalyzes the UG node (with the C → G link, Fig. 4a, top right). CA perturbs the target node strongly since it catalyzes an isolated species, which is only weakly catalyzed, with a strong catalytic link (C → G link)^30^. The perturbation is indeed evident from the change in chemical composition of the network in the bulk aqueous phase—the compositional ranking of the UG and UA species is flipped upon the addition of the CA species which catalyzes the synthesis of UG species (Fig. 4a, bottom right; compare the composition in bottom left with bottom right). While such a disturbance of the composition is expected in a well-mixed solution, to test this in the coacervate compartments, we devised two situations: (a) the network is encapsulated in the coacervate droplet (Fig. 4b), and (b) networks inside the coacervate macrophase (Fig. 4c). When the network was encapsulated inside coacervate droplets and CA encapsulated droplets were used for perturbation, we observed the network composition is protected, i.e., the compartment was indeed able to render protection to the network composition as the added perturbation does not result in a change in the compositional identity (Fig. 4b). Similarly, for the macrophase, perturbation was achieved by adding solution of CA species on top of the separated coacervate macrophase and then incubated. Here too, the coacervate macrophase compartment rendered protection to the compositional identity (Fig. 4c). In the case of coacervate droplets, there is a slight change in the relative proportion of UA and UG in the presence of CA droplets. This could be due to some coalescence between CA and UA, UG-containing droplets or due to diffusion of some surface-bound RNA species which can be easily perturbed by the presence of CA (either from solution or from the surface of CA droplets). However, given the faster timescales of diffusion, the latter could be the most likely cause.

Unlike in surfactant-stabilized aqueous droplets in an oil phase^30^, or vesicles formed by lipids, which are impermeable to large RNA fragments altogether, the network robustness that is conferred by the coacervates is transient—the compartments arise due to a liquid–liquid phase-separation and lack an impermeable boundary. Indeed, upon a longer incubation (4 h compared to 30 min used above) of the system under perturbation, the chemical composition within the coacervate is modified (Supplementary Fig. S11). Perturbation timescales in such condensed coacervate system would depend on multiple parameters like the length of polymers involved, net charge, and structure of the nucleic acids and would require detailed exploration. To be a good model protocell, a coacervate compartment must not only have a robust self-replicating chemistry inside it, but also be able to transport molecules into and out of the compartment. Therefore any associated perturbation to the network robustness can happen on timescales that are long enough for selection to act—one of the possible ways is implementing nonequilibrium scenarios with dynamic fusion and fission of droplets as was recently reported^29^. Altogether, our experiments do indeed show a clear separation of reaction timescales (network identities get perturbed over the course of hours) and diffusion timescales (happening on the timescale of a few minutes as measured from the molecular diffusion), which is crucial for a system to undergo selection and evolve.

## Discussion

Integrating RNA autocatalytic reaction networks with coacervate compartments, brings together four important conceptual frameworks related to the emergence of a protocell from a “primitive” chemical system: (i) the RNA world hypothesis^40–42^ or more broadly, the dynamics of sufficiently long, information carrying catalytic molecules capable of participating in complex reactions and evolving in complexity, (ii) autocatalytic reaction networks, which possess all the attributes required of a simple chemical system to display the properties of robustness, heritability and evolvability^11,18,19^, (iii) dynamic compartmentalization via coacervation^1,2^ which serves to spatiotemporally localize chemistries, and (iv) proposition from Freeman Dyson that the Oparin-like compartments (“coacervates”) could endow self-reproducing reaction networks and undergo Darwinian evolution^43^.

In bringing these various conceptual frameworks together in a single physical realization i.e. coacervate-based compartments with autocatalytic RNA chemistries, our work not only fills a crucial gap of coupling but immediately opens up the possibilities to explore diverse chemical dynamics and evolutionary scenarios: (i) The assembly of catalytic RNAs from smaller inactive RNA fragments in such a charge-rich and condensed physical environment highlights the possibility that the nucleic acid-based self-reproducing systems can achieve fairly complicated functions in seemingly unfavorable conditions. (ii) Cross-catalysis and network formation, not only involves the self-assembly of catalytic molecules but that they can catalyze the synthesis of other network species. (iii) Notably, the enclosed autocatalytic reaction networks assembling RNA ribozymes endow the coacervate compartments with an identity which are protected from perturbations. Such transient protection afforded by the coacervate compartments to these complex ACSs, when coupled with growth and division mechanisms of the compartment^6,29,44^, poises the whole system at a balance between robustness and flexibility, resulting in a heritable, self-reproducing unit, which might itself be considered a minimal protocell. Such a protocell naturally lays the ground for evolutionary mechanisms resembling canonical Darwinian evolution. When compared to vesicles, coacervate compartments are ‘leaky’ due to the lack of a defined boundary and hence using them in any prebiotic dynamical scenario would require that information propagation (e.g., via molecular diffusion) occurs faster than the percolation (replication) of the genetic material they carry. The implementation of hybrid systems based on lipid-coated coacervates, as we have also explored here, could overcome such percolation barriers^45^.

## Methods

### Materials

All the experiments used DNAse/RNase-free water (Thermo Fisher Scientific Product No.: 10977015). Chemicals were purchased from SRL chemicals (Sisco Research Laboratories (SRL) Pvt. Ltd., India) and Sigma Aldrich unless specified otherwise. 4-(2-Hydroxyethyl)-1-piperazinepropane-sulfonic acid (EPPS) was purchased from Alfa Aesar (Product no.: J60511). Two different types of poly(acrylic acid) were used (PAA): high molecular weight HMW-PAA (~>4,000,000 Da, Sigma Aldrich, Product no.: 306231) for preparing coacervates and low molecular weight LMW-PAA (~1800 Da, Sigma Aldrich, Product no.: 323667) for dissolving the coacervates to prepare the samples for gel electrophoresis. Spermine with >97% purity from Sigma (Sigma Aldrich Product No.: S3256) was used for coacervation. 12% denaturing polyacrylamide gels were prepared using acrylamide (SRL chemicals, Product no.: 15657), bis-acrylamide (Sigma Aldrich, Product no.: 146072) in 19:1 ratio and 8 M Urea (Qualigens, Product no.: Q15985) and polymerized using TEMED (tetramethylethylenediamine, SRL chemicals, Product no.: 52145) and ammonium persulfate (SRL chemicals, Product no.: 65553). The 2% agarose gels were prepared from agarose (HiMedia, Product no.: MB002) along with SafeDye stain (SRL Chemicals, Product no.: 53261). Gels were run in 1X Tris-Borate EDTA buffer prepared from Tris (Qualigens, Product no.: Q15965), Boric acid (Fisher Scientific, Product no.: 12005) and ethylenediaminetetraacetic acid (EDTA, Fisher Scientific, Product no.: 12635). Oligonucleotides (DNA primers) were obtained from Sigma Aldrich unless specified otherwise and are mentioned in Supplementary Table S1.

### Methods

#### Transcription of RNAs

All the RNA sequences are the same as used in previous studies^30,37^. RNAs were in vitro transcribed as described previously^35^. For transcriptions, the dsDNA templates were prepared by amplifying the wild-type WXYZ template using specific primers. For PCRs, WXYZ template (at 25 pg/μL) was mixed with 0.5  μM of respective forward and reverse primers (see Supplementary Table S1) in 1X PCR buffer (Thermo Scientific), 0.2 mM dNTPs, 0.02 U/μL of polymerase (Thermo Scientific *Taq* polymerase, Product No.: EP402) and amplified using the following protocol: initial denaturation 94 °C/5 min, then 28 cycles of denaturing 92 °C/1 min, annealing at 57 °C/1 min, extension at 72 °C/1 min and a final extension of 72 °C/5 min. PCR products were isopropanol precipitated by adding 1/10th volume of 3 M sodium acetate pH 5.5 and 1.2 volume of 100% isopropanol to the PCR reaction and centrifuging at 13.8 rcf for 60 min at 10 °C. Pellets were vacuum-dried, resuspended in 20–30 μL of water and used for in vitro transcription reactions as described in ref. ^35^. Briefly, the re-suspended PCR products were mixed with 4 mM of each NTPs, 1X Transcription buffer (Thermo Scientific), 12 mM MgCl_2_, 3 U/μL of T7 polymerase (lab-made), and incubated at 37 °C for 8 h. The transcribed RNAs were purified on 12% denaturing polyacrylamide gels, specific bands were eluted from gels, and isopropanol precipitated in the same way as mentioned above. RNA concentrations were measured using NanoDrop 2000 (Thermo Scientific). The Z fragment RNA used here was custom synthesized by IDT (Integrated DNA Technologies, Belgium) and used without further purification.

#### Formation of PAA-spermine coacervate compartments

In general coacervate compartments were prepared in 25 μL scale volume (unless specified otherwise) by mixing thoroughly at first 5 mM spermine and 25 mM Tris-HCl (pH 8.0) together in water and then adding 12.5 mM of PAA (HMW). Then, 1X ribozyme reaction buffer (AZ Buffer: 30 mM EPPS, 50 mM MgCl_2_, pH 7.0) was added, and the solution was mixed again using pipetting. For the RNA reactions, substrate RNA fragments (after folding) were added prior to spermine addition (see “Methods”). For the coacervate droplets, these compartments were used directly. For the macrophase, the coacervate were settled by centrifuging the tubes at 100 rcf for 20 min at 4 °C (Supplementary Fig. S2). After pipetting out the bulk aqueous phase, the remaining coacervates at the bottom of the tube were used as coacervate macrophase. Both droplets and macrophase were analyzed by microscopy as well as FRAP (see below). To facilitate this, additionally, a 30-nt 5’-Alexa-488-labeled DNA oligonucleotide (custom synthesized from IDT, Integrated DNA Technologies, Belgium, Supplementary Table S1) was added to the coacervate samples, at a 10 nM concentration, prior to the addition of Tris and spermine.

#### Fluorescence recovery after photobleaching (FRAP) measurements

The FRAP measurements were carried out both on the coacervate droplets as well as separated coacervate macrophase (Fig. 1c, d), and samples were prepared as described above. For FRAP, a PDMS (polydimethylsiloxane, SYLGARD^TM^ 184, DOW) sample chamber with a glass cover slip was prepared on which ~100 μL of the sample was added and covered with another glass cover slip. Coacervate droplet samples were kept for 30 min to settle down, whereas separated macrophase samples were used directly after sealing the glass slides with nail polish from the outside (sandwiching between two glass coverslips). Measurements were carried out on the Olympus FV3000 confocal microscope using cellSens software. For each sample, a region of interest (ROI) was photobleached for 10 s using 100% laser power at 488 nm. This is followed by measuring the fluorescence recovery time of ROI. For coacervate droplets (Fig. 2d), ROI circle of diameter 5 μm was chosen, and the time series was acquired at 1.6 fps for 60 s. For separated macrophase samples, the fluorescence recovery time series was acquired at 0.3 fps for 300 s with ROI circle diameter of 18 μm. The fluorescence intensities from the image sequences were measured using Fiji software (https://imagej.net/Fiji,^46^) to generate the recovery curve. Recovery data was normalized using the equation mentioned below, explained in ref. ^22^:

$$F(t)=\frac{[S(t)-B(t)][R(0)-B(0)]}{[R(t)-B(t)][S(0)-B(0)]},$$

where *F*(*t*) is the normalized fluorescence intensity of the ROI at time *t*, *S*(*t*) is sample ROI intensity, *R*(*t*) is reference ROI intensity, *B*(*t*) is background ROI intensity. The data were fitted using the first-order exponential equation given below:

$$f(t)=A(1-\exp (-t/\tau ))+C,$$

where *f*(*t*) represents the normalized fluorescence intensity, *A* represents the amplitude of the recovery, and *C* represents the y-intercept. By fitting the experimental data in this equation, the half-time ${t}_{1/2}=\ln (2)\tau$ was calculated where, *τ* represents the recovery time. The apparent diffusion coefficient (*D*_*app*_) for 2D diffusion was calculated using the equation below^24,47^:

$${D}_{app}=\left(0.88{\omega }^{2}\right)/(4\ln (2)\tau ),$$

where *ω*, represents the ROI radius.

#### Radioactive labeling of RNAs

To facilitate the measurement of the product formation by gel electrophoresis, all the reactions were doped with minor amounts (0.01 μM) of radioactively labeled W or WXY fragment (*γ*^32^*P*). These radioactively labeled RNAs were prepared by mixing 1 μM of RNA with 1X of Shrimp Alkaline Phosphatase buffer (rSAP reaction buffer, New England BioLabs), 1 U/μL of rSAP enzyme (New England BioLabs, Product no.: M0371S), and water in 10 μL reaction scale. Samples were incubated at 37 °C for 40 min and enzyme was heat inactivated at 70 °C for 10 min. The SAP reaction samples were then directly used for kinase reaction by mixing 0.5X of polynucleotide kinase buffer (PNK Buffer A, Thermo Scientific), 5 μL of *γ*^32^*P*-ATP (10 mCi/mL, BRIT Hyderabad, India, Product No.: PLC 101), 0.4 U/μL PNK enzyme (Thermo Scientific, Product No.: EK0031) and water in 20 μL reaction scale. The samples were incubated at 37 °C for 1 h and the reaction was stopped by adding an equal volume of gel-loading buffer (90% formamide, 0.01% xylene cyanol, 0.01% bromophenol blue). Labeled RNAs were purified on 12% denaturing polyacrylamide gels, eluted in 0.3 M of sodium acetate pH 5.5 (2 h at 37 °C) followed by isopropanol precipitation as mentioned above. The pelleted RNAs were resuspened in water and used for the RNA catalysis experiments.

#### Four-fragment self-assembly reactions inside the coacervate compartments

For four-fragment assembly in coacervates, 25 μL coacervate samples were prepared by mixing substrate RNA fragments (W, X, Y, Z at 0.75 μM each) in water, heated at 80 °C/3 min and then incubated 4 °C/3 min (on ice) to fold the RNAs. Then, Tris, spermine and PAA were added in the same way and at the same concentrations as described above followed by addition of 1× ribozyme reaction buffer (30 mM EPPS, 50 mM MgCl_2_, pH 7.0). The solution was mixed thoroughly by pipetting. For the macrophase, bulk of the aqueous phase was removed from the top and the settled condensed phase at the bottom of the tube was incubated at 48 °C (Supplementary Fig. S2). In the case of coacervate droplets, the solution was incubated at first and then to analyze the product formation from the droplets, coacervates were separated from the bulk solution in similar way as mentioned above. In each case, for the time courses, a separate reaction tube was prepared for each time point. For preparing samples for the gel analysis, after centrifugation and separating the bulk of the solution, 25 μL water was added to the separated coacervates, followed by 25 μL of PAA-stopping solution (50 mM PAA-LMW, 166 mM EDTA, 333 mM NaOH) and the samples were then vortexed vigorously. Then from this mixture, 20 μL was mixed with 20 μL of stopping solution (90% formamide, 50 mM EDTA, 0.01% xylene cyanol, 0.01% bromophenol blue) and analyzed on 12% denaturing polyacrylamide gels. For the bulk aqueous phase control, the reactions were carried out without any of the coacervate reagents (PAA, Spermine, Tris), however, the time points were collected and processed in the same way as the coacervate samples.

#### Lipid-vesicle coating of coacervate droplets and catalysis

The small unilamellar vesicles (SUVs) were prepared by using a previously reported protocol^48^. Briefly, 25 μL of DOPC (1,2-dioleoyl-sn-glycero-3-phosphocholine, 25 mg/mL prepared in chloroform; Avanti Polar Lipids) and 5 μL of Liss-Rhod-PE (1,2-dioleoyl-sn-glycero-3-phosphoethanolamine-N-(lissamine rhodamine B sulfonyl), 10 μg/mL prepared in chloroform; Avanti Polar Lipids) were mixed in a 2 mL Eppendorf tube, vacuum-dried overnight followed by drying under Nitrogen before resuspended in 1 mL nuclease-free water. To prepare SUVs, a probe sonicator (VC 750 (750W), stepped microtip with diameter 3 mm) with an on/off pulse of 1.5 s/1.5 s and 32% of the maximum sonicator amplitude were used. The tube was placed in an ice bath in between the sonication steps (sonication for 30 s with a 1 min gap in between). Then the tube was briefly spun (using micro-centrifuge) and SUVs were stored at 4 °C. To carry out four-fragment assembly of WXYZ catalyst inside the vesicle-coated coacervate droplets, at first, a coacervate solution was prepared as mentioned above by adding all the four RNA fragments (W, X, Y, Z) at 0.75 μM each (after folding together), 5 mM spermine and 25 mM Tris-HCl (pH 8.0) in water. Then 12.5 mM of PAA (HMW) was added, solution was mixed thoroughly by pipetting followed by addition 1X ribozyme reaction buffer (AZ Buffer: 30 mM EPPS, 50 mM MgCl_2_, pH 7.0) and again mixing by pipetting. Finally, 4 μL of SUVs solution was added to 25 μL coacervate solution, mixed by pipetting, and kept on ice for 5 min to allow for coating. Then to initiate the reaction, the solution was incubated at 48 °C. As mentioned above, a separate reaction tube was prepared for each time point and samples were processed in the same way without any additional steps.

To measure the stability of vesicle-coated coacervate droplets, their size distribution was analyzed over time. Lipid-vesicle-coated coacervate droplets were prepared in the same way as mentioned above, incubated at 48 °C, and analyzed under the microscope. The images were captured in the fluorescent channel of an inverted microscope (100X oil objective, exposure time 100 ms) and the droplet sizes were calculated using the “*analyze particle*” function in Fiji software (https://imagej.net/software/fiji/). To analyze the leakiness or diffusion of oligonucleotide from the bulk phase into vesicle-coated coacervate droplets, droplets were prepared with a 30-nt long fluorescently labeled DNA oligonucleotide (Alexa-488-labeled, same as used in Fig. 1, see Supplementary Table S1), both alone (Fig. 2d, top) as well as hybridized to the ~200-nt-long WXYZ RNA (Fig. 2d, bottom), and then coated with DOPC vesicles (without doping with Liss-Rhod-PE in order to avoid bleed through of fluorescence signal). For hybridization, 5 nM of 30-nt long fluorescently labeled DNA oligonucleotide was mixed with 50 nM of WXYZ RNA (1:10 ratio) and hybridize by heating the solution at 80 °C for 3 min and cooling down on the ice for 3 min. This hybridized oligonucleotide mixture was used for preparing the coacervate sample. Then the sample was incubated at 48 °C and images were captured in the fluorescent channel of an inverted microscope (100X oil objective, exposure time 100 ms) at 0 h and at 4 h. The fluorescent images were analyzed using Fiji software (https://imagej.net/software/fiji/) for the change in fluorescence intensity inside the vesicle-coated coacervate droplets. To analyze the images, a line was taken through the center of the droplet on the raw image (without any pre-processing of the images) and fluorescence intensity profile was extracted along that line. For each sample and the time point, ~10 droplets of approximately similar sizes were analyzed and the average fluorescence intensity of several droplets as a function of distance from the center of the droplet was plotted (Fig. 2d).

#### Measuring autocatalytic rate constants inside the coacervate macrophase

Effect of catalyst and autocatalytic rate constants inside the coacervates were measured using the same strategy as developed by von Kiedrowski^49^ and used for the bulk reactions in the previous studies^30,31^ by analyzing two-fragment self-assembly reaction (between WXY and Z RNAs). However, here low concentration of substrate RNAs and WXYZ catalysts were used. Briefly, coacervate samples were prepared as described above using 0.1 μM of WXY (along with 0.01 μM ^32^*P* radioactive labeled WXY) and Z RNAs each along with different concentration of seeded WXYZ catalyst (at 0, 0.015, 0.15, 0.3, 0.5 μM, having same IGS as WXY fragment). To measure initial rate of reaction, samples were incubated for a very short period of time (~12 min) and reactions were stopped to prepare gel samples in the same way as described above. Initial rates of formation of covalent WXYZ were derived from the time courses of assembly of WXY and Z fragments (only from the linear portion of the time courses) in the presence of different concentrations of seeded product (WXYZ) catalyst added at the beginning of the reaction. As observed earlier^30,31^, the initial rate of formation of WXYZ (*r*_0_) was found to depend linearly on the concentration of the seeded WXYZ. This dependence can be described using the following relationship:

$${r}_{0}={k}_{a}[x]+{k}_{b},$$

where *x* is the concentration of the covalent ribozyme, *k*_*a*_ is obtained from the slope and *k*_*b*_ is obtained from the y-intercept. Here, *k*_*a*_ quantifies the formation of WXYZ product synthesis by covalent WXYZ ribozyme and *k*_*b*_ quantifies the formation of WXYZ product synthesis by non-covalent ribozyme assembly (trans-catalysis by the non-covalent complex of WXY and Z fragments^36,37^). For the aqueous phase control (bulk reaction without any polymers), due to low reaction rates in the absence of coacervation, self-assembly reactions were carried for the longer time period (up to 90 min) and higher concentration of WXYZ catalyst (at 0, 0.5, 0.75, 1.0, 2.0 μM were added at the beginning of each reaction. Rest of the processes were done in a similar manner as for the coacervate samples.

#### Network formation inside the coacervate compartments

To confirm the formation of the network inside the coacervates, respective RNA fragments to construct two-, three- and, four nodes networks were mixed together at 0.5 μM each along with an equivalent amount of Z RNA fragment, and 0.01 μM of ^32^*P*-labeled WXY RNA fragment for UU in water at 25 μL scale. The samples were then heated at 80 °C/3 min and then incubated 4 °C/3 min (on ice) to fold the RNAs and coacervation was induced as described above. The coacervate droplets were directly incubated at 48 °C (for 1 h) and then separated post-incubation for the polyacrylamide gel analysis, whereas the coacervate macrophase was separated from bulk of the solution (as above, Supplementary Fig. S2) and incubated at 48 °C for 1 h. Time points and gel analysis was done in the same way as described above in the four-fragment assembly section.

#### Network composition measurements

To measure the network composition inside coacervates, WXY RNA fragments for the respective nodes at 0.5 μM each along with an equivalent amount of Z RNA fragment were used. For measuring amount of different nodes from the same network, multiple reactions (equal to the number of nodes to be measured) were set-up in parallel where each one is doped with 0.01 μM of the respective ^32^*P*-labeled WXY RNA fragment. For example, in the case of two nodes network (UU, AA), two parallel reactions were set-up where one reaction was doped with ^32^*P*-labeled UU WXY RNA fragment and another one was doped with ^32^*P*-labeled AA WXY RNA fragment. After adding all the RNAs, the samples were then heated at 80 °C/3  min and then incubated 4 °C/3 min (on ice) to fold the RNAs, followed by inducing coacervation as described above. The coacervate droplets were directly incubated at 48 °C (for 30 min) and then separated post-incubation for the polyacrylamide gel analysis, whereas the macrophase was separated from bulk of the solution (as above) and incubated at 48 °C for 30 min. Time points and gel analysis were done in the same way as described above in the four-fragment assembly section.

#### Network perturbation

In order to study network perturbation, at first coacervate solution containing WXY fragments of UA and UG nodes at 0.5 μM each along with an equivalent amount of Z RNA fragment was prepared in a similar way as mentioned in the section above (Network composition). For the droplet phase perturbation, a separate coacervate droplet population containing 0.5 μM of WXY fragment for CA along with 0.5 μM of Z RNA fragments was prepared and mixed in 1:1 ratio with coacervate droplets containing UA and UG. The solution was mixed well by pipetting and incubated at 48 °C. For the perturbation in coacervate macrophase, after separating the condensed phase containing the WXY fragments of UA and UG from the bulk of the solution, 0.5 μM of WXY fragment for CA along with 0.5 μM of Z RNA fragments were added on the top of the coacervate macrophase (~4 μL) and then samples were incubated 48 °C. For aqueous phase control, the reactions were carried out without any of the coacervate reagents (PAA, spermine, Tris-buffer); however, the time points were collected and processed in the same way as the coacervate samples.

### Reporting summary

Further information on research design is available in the Nature Portfolio Reporting Summary linked to this article.

## Supplementary information

Supplementary Information

Reporting Summary

## Data Availability

The data that support the findings of this study are available from the corresponding authors upon reasonable request.
