# Supplementary material for: Multispecies autocatalytic RNA reaction networks in coacervates
Source: Commun Chem. 2023 May 8;6:91. doi: 10.1038/s42004-023-00887-5 (PMC10167250; doi:10.1038/s42004-023-00887-5)
Supplement: Supplementary file 1 — Supplementary Information [file 42004_2023_887_MOESM1_ESM.pdf]

Supplementary Information: Multispecies autocatalytic RNA reaction networks in coacervates

Sandeep Ameta,[1,*] Manoj Kumar,[1] Nayan Chakraborty,[1] Yoshiya J. Matsubara,[1] Prashanth S,[1] Dhanush Gandavadi,[1] and Shashi Thutupalli[1,2,†]

[1] *Simons Centre for the Study of Living Machines, National Centre for Biological Sciences, Tata Institute of Fundamental Research, Bengaluru, India*

[2] *International Centre for Theoretical Sciences, Tata Institute of Fundamental Research, Bengaluru, India*

[*] sandeep.ameta@ashoka.edu.in; Present address: Trivedi School of Biosciences, Ashoka University, Plot No. 2, Rajiv Gandhi Education City, P.O. Rai, Sonepat, Haryana-131029, India

[†] shashi@ncbs.res.in

Supplementary Notes

Note 1: Autocatalytic nature of the WXYZ assembly and effect of phase-separation

Azoarcus ribozyme self-assembles from its fragments (two, three, or four) in an autocatalytic manner [1, 2]. The autocatalytic nature of the reaction can be observed from the sigmoidal nature of the covalently self-assembled product formed as a function of time (as observed in Fig. 2). In the case of *Azoarcus* system, though the sigmoidal curve can be easily observed for the four-fragment assembly [1], it is not so straightforward for the two-fragment assembly, owing to its faster reaction timescale [2–4]. This can somewhat be resolved by slowing down the reaction either by lowering the concentration of substrate or magnesium. Even though the sigmoidal nature of the curve is a consequence of the autocatalytic nature of the reaction, a better demonstration is through the seeding experiments, where the substrate of fixed concentration is seeded with varying catalyst concentrations (product of the reaction) added at the starting of the reaction [5]. In such an experiment, the positive feedback is confirmed if there is an increase in the rate of reaction as a function of (seed) catalyst concentration. We have observed a clear positive effect of the catalyst in the seeding experiment for the assembly of the *Azoarcus* ribozyme (Supplementary Fig. S6). In addition, we have also observed the positive effect of catalyst on the four-fragment assembly of the *Azoarcus* ribozyme (Supplementary Fig. S4).
Besides the qualitative observation of the positive feedback, such measurements also allow quantifying autocatalytic rate constants, k_a (feedback from the product of the reaction, *i.e.* covalent WXYZ catalyst) and k_b (background reaction, *i.e.* catalysis by non-covalent WXYZ complexes). Please note that the catalysis by non-covalent complexes is due to the formation of the active catalytic pocket formed even in the fragmented version of the ribozyme [2, 4] and not due to the spontaneous background (uncatalyzed). While in literature [5, 6], k_b accounts for the reaction carried out due to the spontaneous reaction, for simplicity, in the case of *Azoarcus* ribozyme assembly we use k_b for the catalysis by non-covalent WXYZ complexes. Therefore in the case of *Azoarcus* ribozyme, there are two catalytic components – covalent WXYZ and non-covalent complexes. In the initial phase of the reaction, due to the higher concentration of RNA fragments, the non-covalent complexes form really fast (<1 *min*) and majorly govern the self-assembly process [3, 4]. This is more evident in the phase-separated coacervate compartments as they tend to concentrate biomolecules inside them (Supplementary Fig. S8) such that the effect of k_b is more pronounced on the assembly process of the *Azoarcus* ribozyme. This is also captured in our measurements (Supplementary Table S2 and Fig. S6), and as a consequence, the formation of autocatalytic reaction networks is indeed affected by the catalytic activity of non-covalent complexes, k_b in the phase-separated compartment rather than k_a.

Note 2: Rate enhancement inside the coacervates

Another notable feature of the reactions inside the coacervate compartments is the rate enhancement of the assembly, as also observed earlier for other catalytic RNAs [7]. When compared with the same self-assembly reaction in the absence of coacervation (bulk control), the formation of the assembled ribozyme product is significantly faster in coacervate compartments, evident by a shorter 'lag-phase' (Fig. 2F). Reactions within the coacervate macrophase are faster than those in coacervate droplets — this is to be expected since the surface area to volume ratio of the droplets is much higher than that for the macrophase which prevents any dynamic exchange with the bulk that can slow the effective rates of the reactions.

These reaction rate enhancements are evident in the kinetic parameters (Supplementary Table S2) obtained from fits of the model to the data: while the rates of the background reaction k_b in the coacervate compartments are significantly higher than those in the bulk phase, the reaction rate catalyzed by the covalent catalyst k_a does not indicate such an enhancement. A similar enhancement is observed in the initial production rate measured in WXYZ assembly from two fragments (Supplementary Fig. S6). Spermine, which is present abundantly in the coacervate phase is known to stabilize RNA structures [8], could lead to rate enhancements that observed here; controls where reactions were carried out with and without spermine, however, rule out that possibility as similar amount of product formed in both the cases (Supplementary Fig. S7). Instead, we find that the increase in the reaction rates is attributable to the increase of the RNA concentration within the coacervate phase — indeed, we observe a ~ 50 fold increase in the RNA amount inside the coacervate macrophase phase compared to that in the aqueous phase alone (Supplementary Fig. S8).

Note 3: Perturbation scenario in coacervate compartments

Studying the perturbation scenarios in self-replicating chemistries is crucial in developing an evolvable protocell as one of the significant roles of the compartment is to protect the identity of the encapsulated replicating system. In the case of ACSs, the network composition are amenable to the perturbations which can occur due to spatial and temporal heterogeneity of the prebiotic environment, or due to the presence of parasitic/competing species. Furthermore, it has been (theoretically) shown that slight exchange between compartments or with the environment affects the maintenance of self-replicating systems, particularly ribozyme formation using substrate fragments (Kamimura A. *et al.* [9]). While the notion of parasitic species is not clear in the autocatalytic reaction networks, especially the cooperative networks formed by *Azoarcus* ribozymes [3], perturbation due to environmental factors or the presence of competing catalysts can be conveniently studied.

In the current study, we explore one such scenario where the presence of coacervate compartment renders robustness to the network composition (therefore *compositional identity*) against a competing catalyst (Fig. 4). The perturbation experiment is designed to mimic a situation in which a network (with node UA and UG) is already inside the coacervate compartment and is confronted with a competing species (CA) from the environment which targets the isolated node (UG). Without perturbation, UA dominates over UG as there is no node catalyzing the synthesis of UG (apart from weak G→U wobble link [10]. While in the bulk condition, the presence of CA perturbation changes the ranking of UA and UG, the network composition is found to be preserved in both coacervate droplets as well as macrophase (Fig. 4). Indeed due to the membraneless nature of coacervates, the CA would ultimately diffuse into the compartments and start perturbing the network composition, however, at sufficiently longer timescales than the diffusion of molecules (Supplementary Fig. S11). Such experiments highlight that there is a timescale regime under which even membraneless phase-separated coacervate compartments can provide robustness to the networks.

TABLE S1. Sequences of all the oligonucleotides used in the study.

#	Description	Oligonucleotide sequence
1	Forward primer to prepare template for WXY RNA fragment with 'GUG' as IGS	CTGCAGAATTCTAATACGACTCACTATAGTGCCTTGCGCCGGGAAACCACGC
2	Forward primer to prepare template for WXY RNA fragment with 'GAG' as IGS	CTGCAGAATTCTAATACGACTCACTATAGAGCCTTGCGCCGGGAAACCACGC
3	Forward primer to prepare template for WXY RNA fragment with 'GCG' as IGS	CTGCAGAATTCTAATACGACTCACTATAGCGCCTTGCGCCGGGAAACCACGC
4	Reverse primer to prepare template for WXY RNA fragment with 'CAU' as tag	ATGTGCCTTAGGTGGGTGC
5	Reverse primer to prepare template for WXY RNA fragment with 'CUU' as tag	AAGTGCCTTAGGTGGGTGC
6	Reverse primer to prepare template for WXY RNA fragment with 'CGU' as tag	ACGTGCCTTAGGTGGGTGC
7	Forward primer to prepare template for W RNA fragment	same as primer #1
8	Reverse primer to prepare template for W RNA fragment	ATGCGGGCGCTTAGGTTTCG
9	Forward primer to prepare template for X RNA fragment	TAATACGACTCACTATAGGCATCGGGCGTATGGCAACGCCG
10	Reverse primer to prepare template for X RNA fragment	ATGGCGCCGAAGCTTGGC
11	Forward primer to prepare template for Y RNA fragment	TAATACGACTCACTATAGGCATGCGCCGATGAAGGTG
12	Reverse primer to prepare template for Y RNA fragment	ATGTGCCTTAGGTGGGTGC
13	Forward primer to prepare template for WXYZ catalyst	same as primer #1
14	Reverse primer to prepare template for WXYZ catalyst	CCGGTTTGTGTGACTTTCGCC
15	30nt oligonucleotide used for the FRAP experiments (custom synthesized)	/5Alex488N/CCGGTTTGTGTGACTTTCGCCACTCCCTGG
16	Z RNA sequence (custom synthesized)	rGrGrCrArUrCrGrCrUrArUrGrGrUrGrArArGrGrCrArUrArGrUrCrCrAr GrGrGrA rGrUrGrGrCrGrArArArGrUrCrArCrArCrArArArCrCrGrG

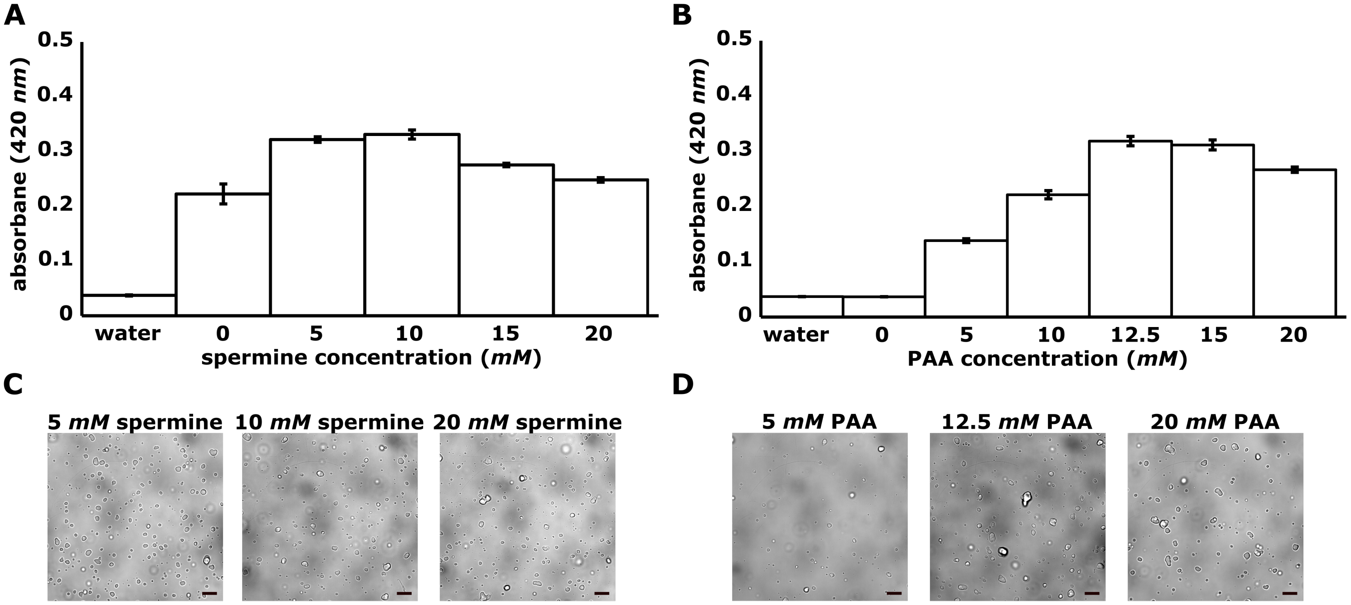

FIG. S1. Turbidity assay. Bar graph showing turbidity measured for coacervate solutions at different polymer concentrations to know the dynamic range of coacervation process. Turbidity was quantified by measuring the absorbance of the solution at 420 *nm* wavelength. **A**. At fix concentration of PAA (12.5 *mM*) and varying spermine concentrations. **B**. At fix concentration of spermine (5 *mM*) and varying PAA concentrations. All the other conditions of coacervation were kept same as mentioned in Material and Methods. **C, D**. Bright-field microscopy images of PAA-spermine coacervate droplets' samples from the turbidity assay. C. At a fixed concentration of PAA (12.5 *mM*) and varying spermine concentrations (5, 10, 20 *mM*). D. At a fixed concentration of spermine (5 *mM*) and varying PAA concentrations (5, 12.5, 20 *mM*). The scale bar is 10 μm for all the images.

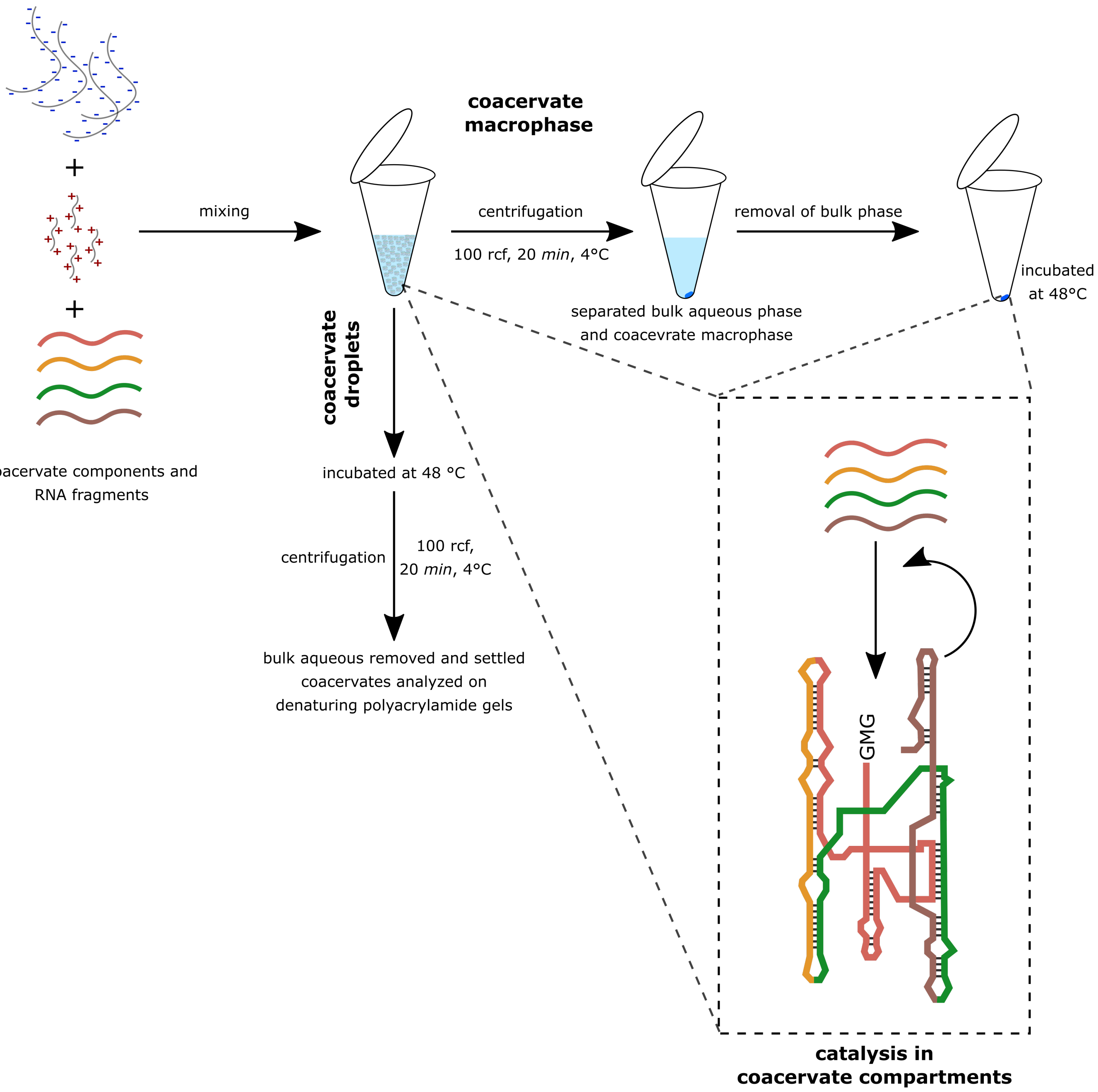

FIG. S2. Protocol for analyzing the WXYZ assembly in the coacervate compartments. Schematic of the protocol showing the steps to prepare coacervates and measuring catalysis inside the coacervate droplets and coacervate macrophase. Briefly, all the components of forming coacervates including RNAs are mixed together thoroughly (see Material and Methods) to form coacervates. For the catalysis inside coacervate droplets, the solution was directly incubated at 48°C and then after the reaction, coacervate phase was separated from the bulk of the solution by brief centrifugation (100 rcf, 20 *min*), the bulk phase was discarded, and the settled coacervate phase was processed for the gel analysis. For the catalysis in coacervate macrophase, the centrifugation was done prior to the incubation and after removing the bulk liquid phase, the settled coacervate macrophase at the bottom of the tube was incubated at 48°C, and then processed for the gel analysis in the same method as described for droplets.

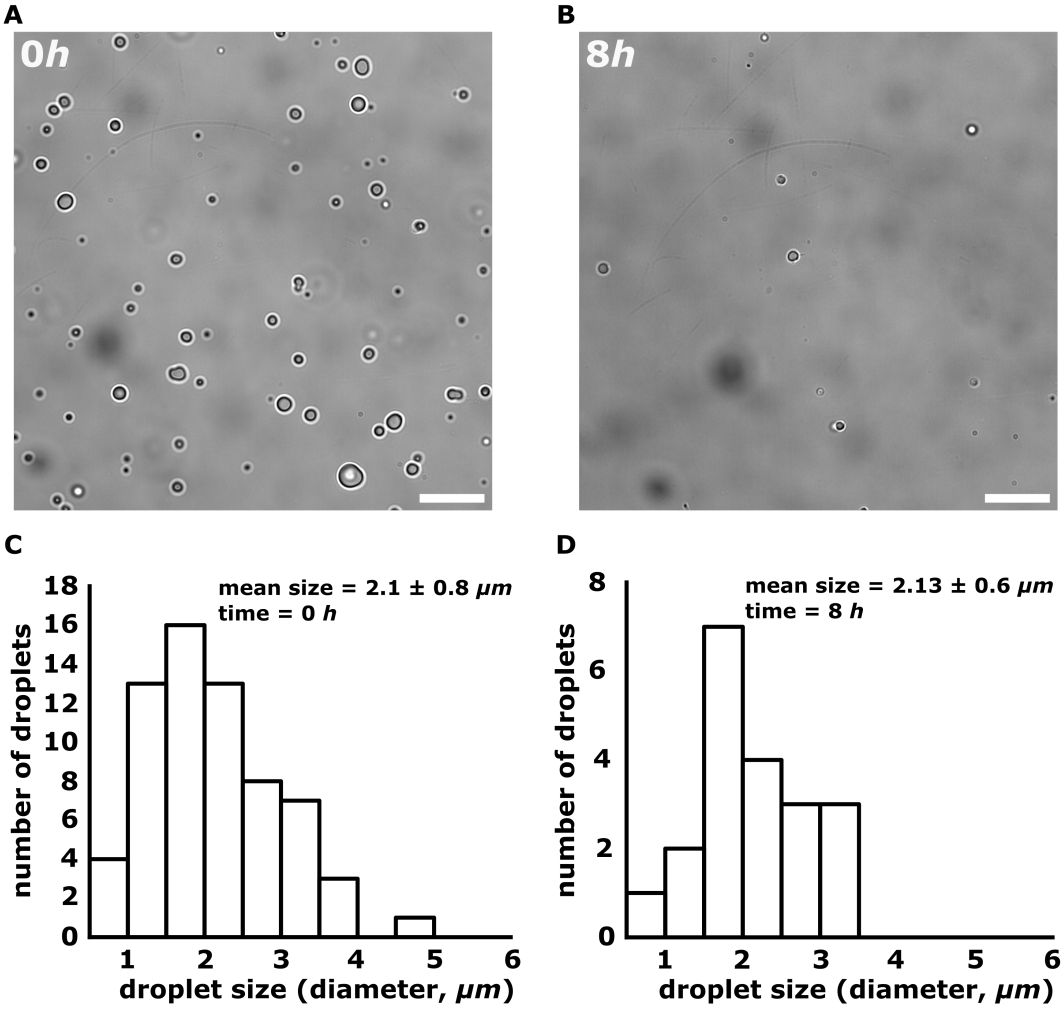

FIG. S3. Coacervate droplet stability. Bright-field microscopy images and size distribution of coacervate droplets (without vesicle coating) to demonstrate that droplets are stable even for longer time periods. **A**. Image at 0 *h*. **B**. Image at 8 *h*. **C**. Size distribution plot for droplets at 0 *h* (n = 60). **D**. Size distribution plot for droplets at 8 *h* (n = 20). The result shows that droplets are stable and visible even after incubation at 48°C for longer periods. Though the density of droplets decreased as they settled down at the bottom of the tube, the mean size remain unaffected. Scale bar is 15 μm for both images.

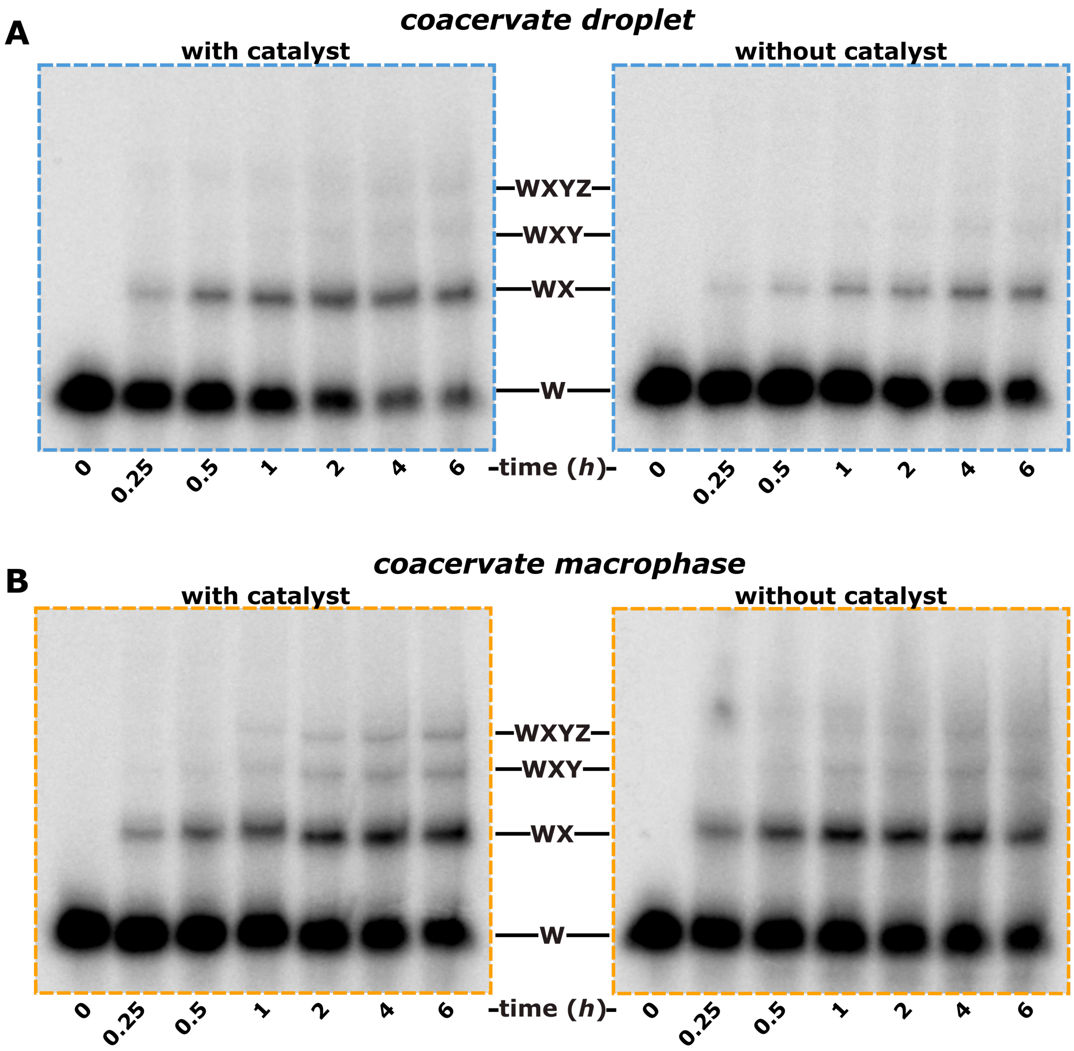

FIG. S4. Positive feedback from the product of the reaction on the assembly of WXYZ. Polyacrylamide gel images showing the effect of catalyst (covalent ribozyme, product of the reaction) on its own assembly from the inactive RNA fragments W, X, Y, and Z inside coacervate droplets (**A**) as well as macrophase (**B**). For both case, assembly is carried out in the presence (*left*) and absence (*right*) of the catalyst (full-length covalent WXYZ ribozyme, 0.5 μM). The catalyst is added together with the RNA fragments (0.1 μM each) at the start of the reaction.

TABLE S2. Kinetic modelling parameters.

**Conditions**	**a (*h*$^{-1}$)**	**b (*h*$^{-1}$)**	**c**	**k_a (*min*$^{-1}$)**	**k_b (*nMmin*$^{-1}$)**
Bulk reaction	1.0 ± 0.1	0.0020 ± 0.0006	0.16 ± 0.01	0.0027 ± 0.0003	0.00057 ± 0.00017
Coacervate droplets	0.96 ± 0.25	0.010 ± 0.003	0.13 ± 0.02	0.0021 ± 0.0006	0.0022 ± 0.0008
Coacervate macrophase	1.2 ± 0.3	0.03 ± 0.01	0.078 ± 0.006	0.0015 ± 0.0004	0.004 ± 0.001
Coacervate droplets (fitting up to 2h)	3.4 ± 0.9	0.003 ± 0.002	0.053 ± 0.007	0.0030 ± 0.0009	0.0003 ± 0.0002
Coacervate macrophase (fitting up to 2h)	2.9 ± 0.7	0.015 ± 0.005	0.056 ± 0.003	0.0027 ± 0.0006	0.0014 ± 0.0005

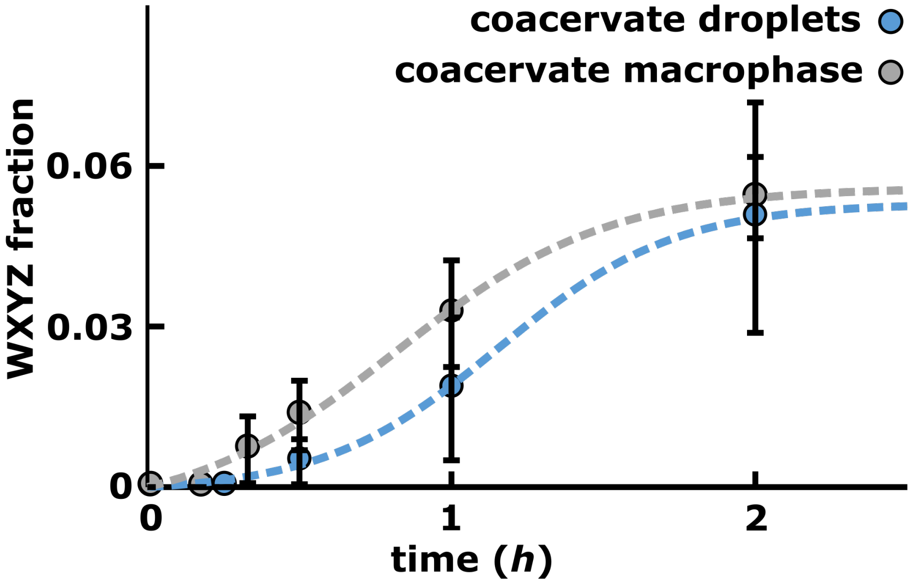

FIG. S5. Kinetic modelling fit for the early part of the reaction.

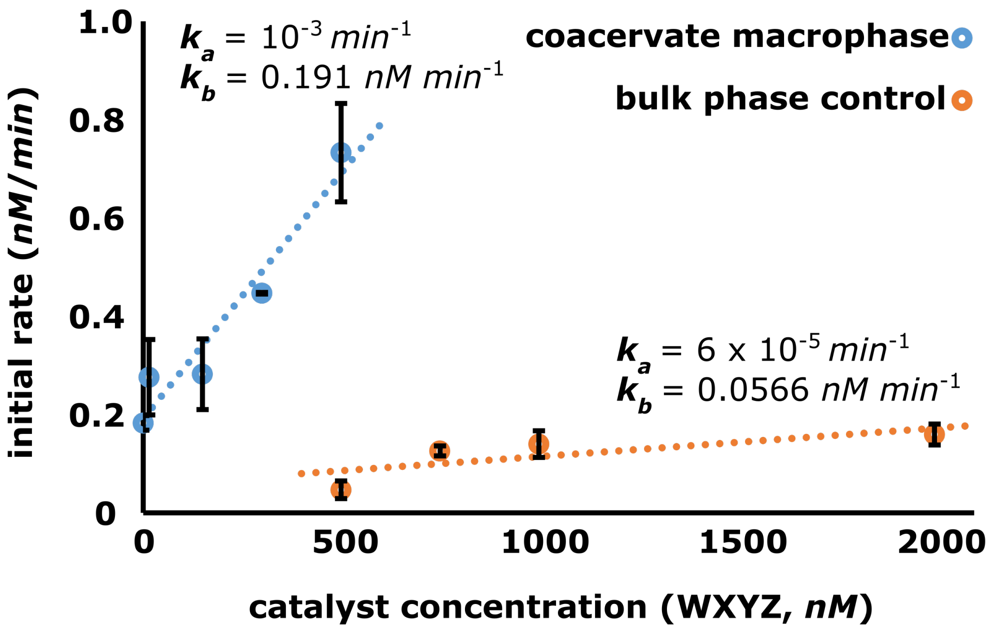

FIG. S6. Positive feedback from the product of the reaction. Graph showing the dependence of the initial rate of WXYZ formation on the different amounts of WXYZ catalyst (product of the reaction) seeded at the start of the reaction. Each data-point on the graph represents the initial rate derived from the time-courses in presence of the different amounts of WXYZ. The autocatalytic rate constants are derived in the same way as reported earlier [2, 4, 10]. The dashed line in the data points represents a linear fit with r^2 of 0.94 for coacervate macrophase and 0.59 for the bulk phase control. Derived k_a and k_b are shown in the inset. The experimental details are mentioned in Material and Methods.

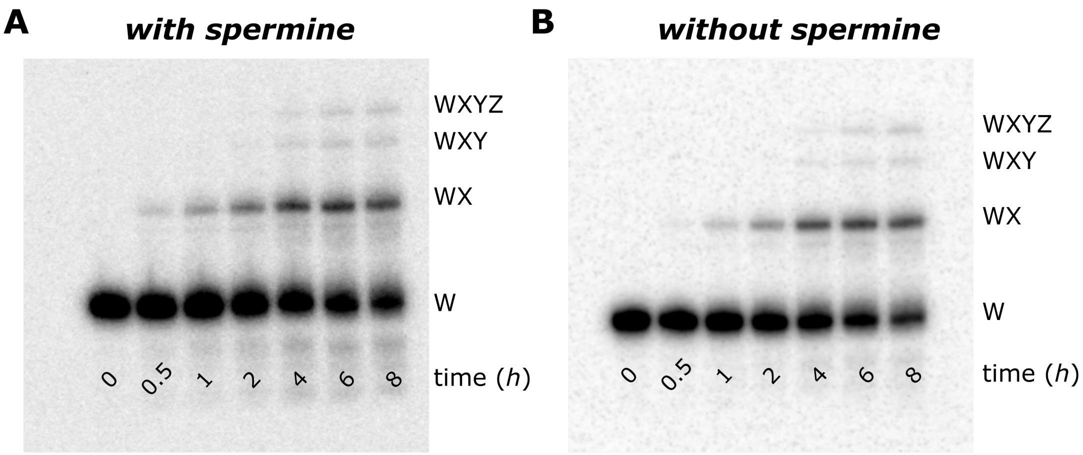

FIG. S7. No effect of spermine on self-assembly of WXYZ. Polyacrylamide gel images showing that spermine has no effect on the self-assembly of substrate RNA fragments (W, X, Y, Z) to form WXYZ ribozymes. Here self-assembly reactions are carried out with 0.75 μM each W, X, Y and Z RNA fragments in presence of 5 mM spermine (**A**) and in absence of spermine (**B**).

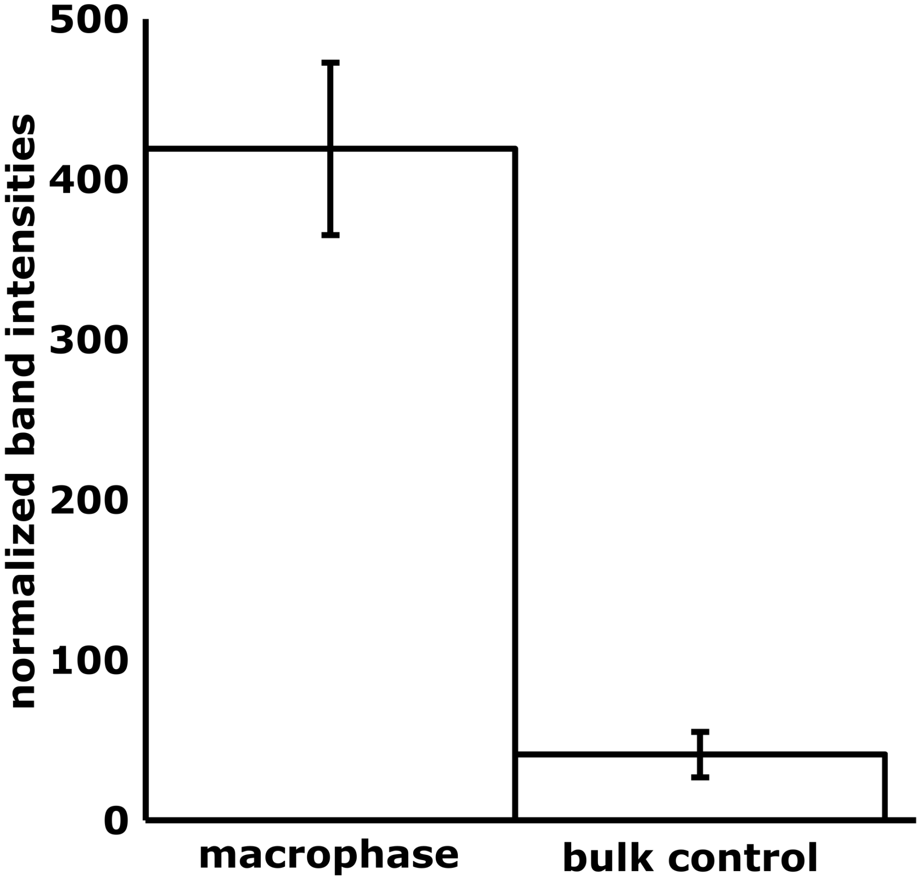

FIG. S8. Increase in the RNA amount inside the coacervate macrophase. Bar graph showing the increase in RNA amount inside the coacervate macrophase ($\sim$ 50-fold) compared to the bulk phase. To measure the enhancement, samples containing W, X, Y, and Z (0.75 μM each) RNA fragments along with spiked amount of radioactive ^{32}P-labeled W fragment (0.01 μM) were prepared with polymers and separated as mentioned above. The bulk, as well as coacervate phase, were then analyzed on polyacrylamide gels and normalized band intensities are plotted here. For the analysis, settled coacervate macrophase was resuspended in the same amount of water as the bulk sample volume. Then an equal volume of both (resuspended coacervate macrophase and bulk control) samples were loaded on the gel (after adding the same amount of PAA stopping and gel loading solutions, see Methods section). After extracting the band intensities, coacervate macrophase band intensities were multiplied by the volume of the water added to equalize the sample volume. All the measurements are done in triplicates and the mean is plotted along with the standard deviation. The results indicate that there is $\sim$ 50-fold increase of RNA concentration inside the coacervate corresponding to 37.5 μM of RNA (effective) concentration.

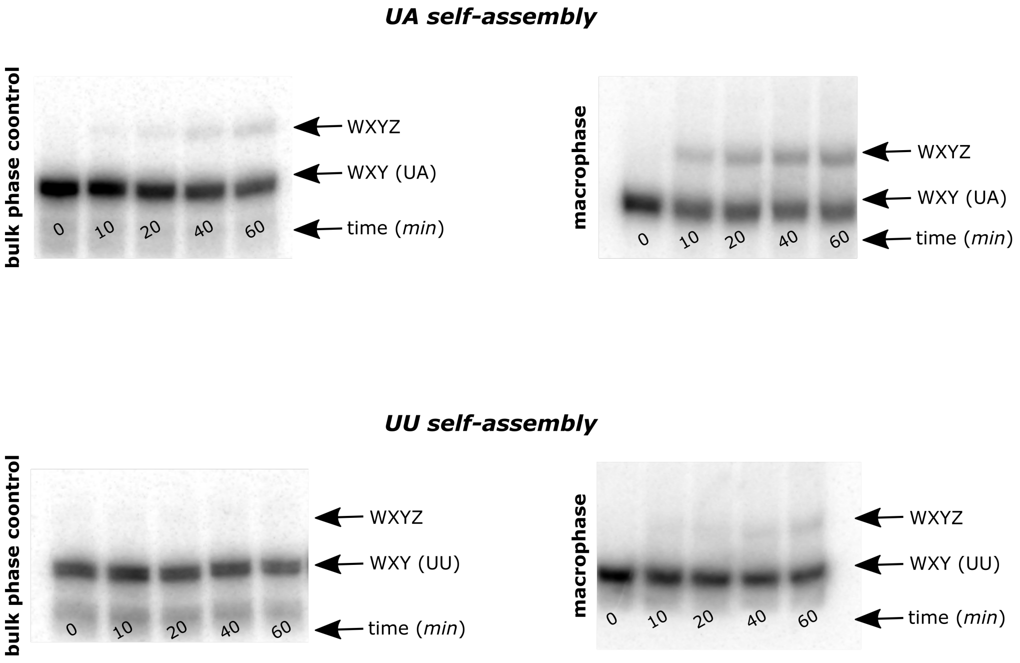

FIG. S9. Poor and good self-assembling WXYZ ribozyme inside the coacervate macrophase. Polyacrylamide gel images showing the product formation for a good self-assembler (UA) in bulk (*top left*) and in the coacervate macrophase (*top right*). The product formation is significantly lower for a poor self-assembler (UU) in bulk (*bottom left*) and even in condensed coacervate macrophase (*bottom right*). Here, two-fragment assembly is carried out between WXY and Z fragments (0.5 μM each) of UA and UU in presence of spike amount of respective radioactive ^{32}P-labeled WXY RNA fragment (0.01 μM). Samples are analyzed on a 12% denaturing polyacrylamide gels (see Material and Methods).

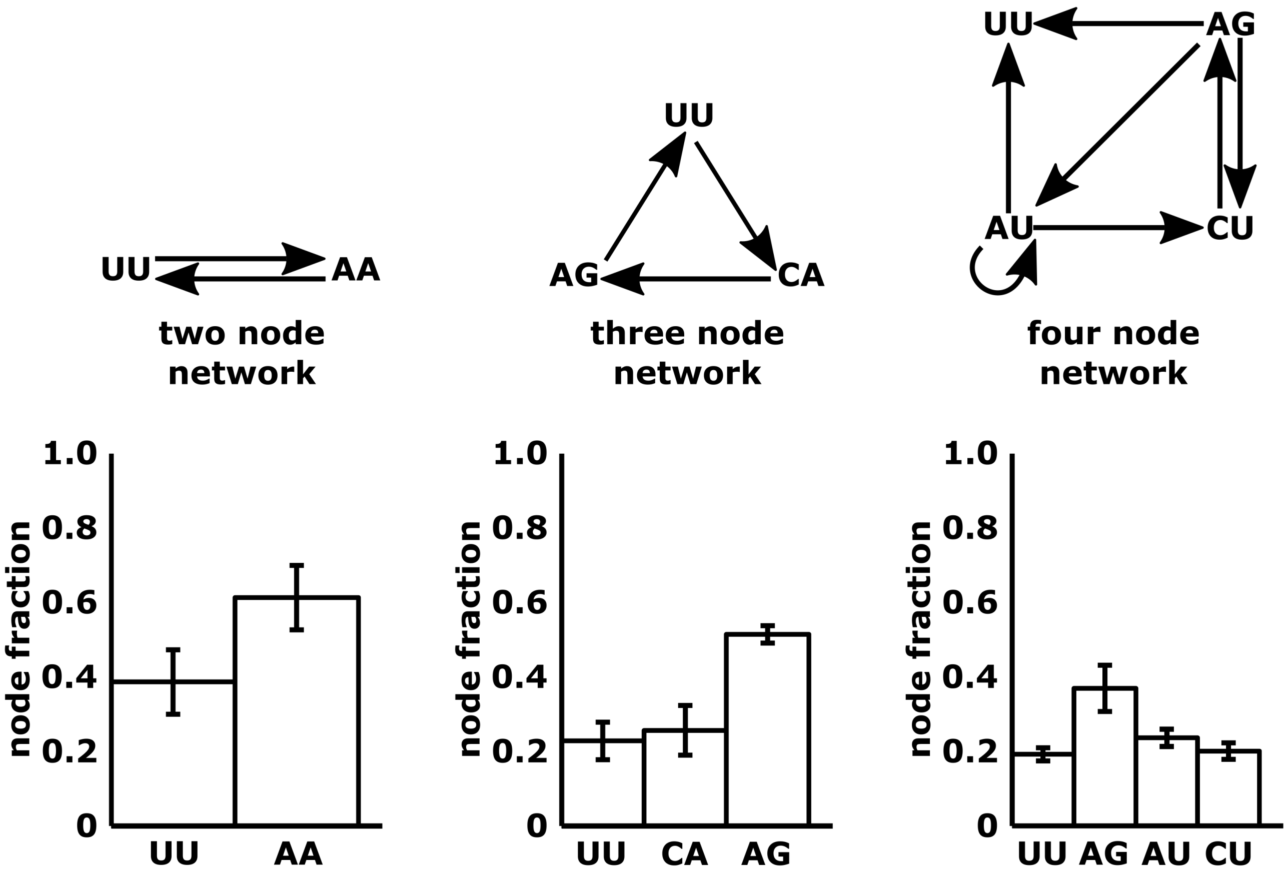

FIG. S10. Network compositions of two, three, and four nodes network in absence of any polymer in the bulk aqueous phase. Network structures are shown on *top*. Bar-graphs showing the measured composition (*i.e.*, relative fraction of individual ribozyme species in the network) of the networks for two-node (*left column*), three-node (*middle column*), and four-node networks (*right column*). All the measurements are done in triplicates and mean WXYZ product formation is plotted as fraction w.r.t. to each node along with standard deviation. See Material and Methods for the experimental procedure.

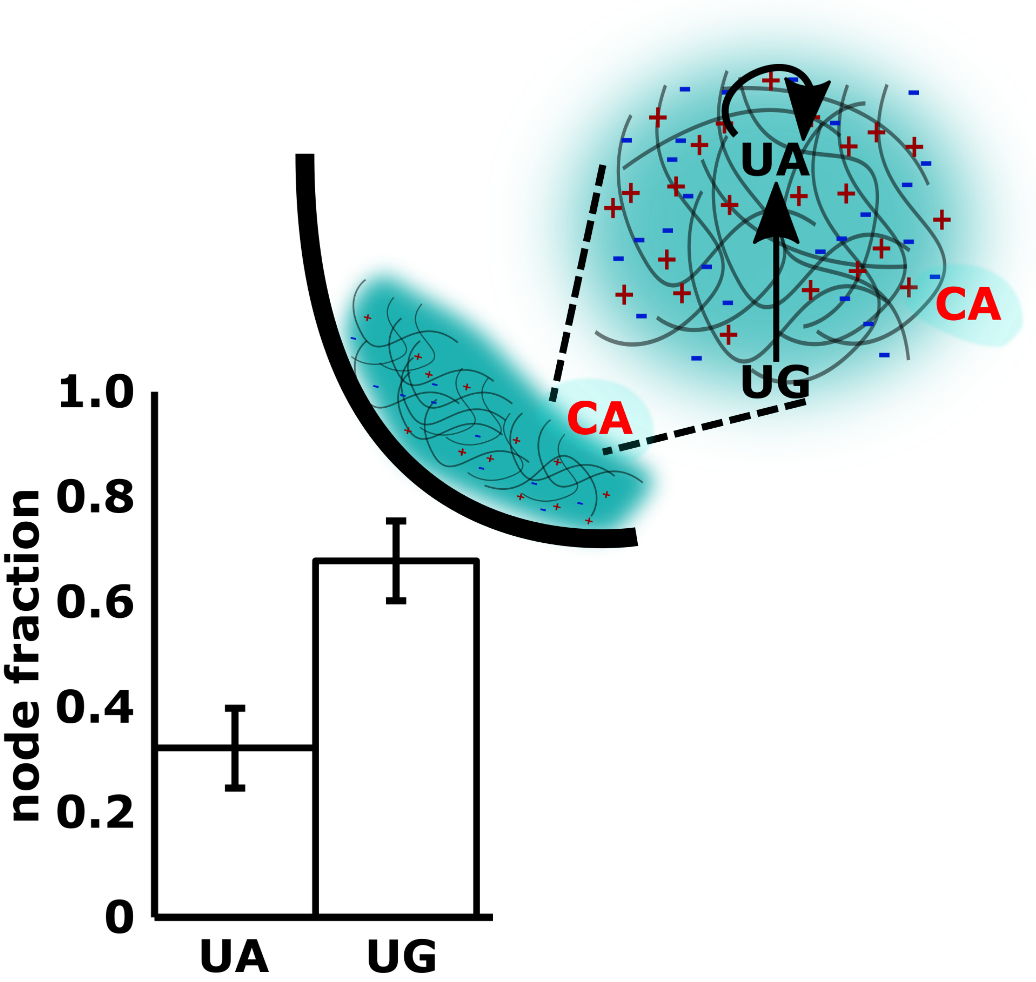

FIG. S11. Transient compositional robustness of cross-catalytic network in the coacervate macrophase. The composition of a two-node network is subjected to perturbation by adding a strong catalyzing node ('CA', due to C→G link, see [4]) after encapsulating the network inside the coacervate macrophase. This is a similar experiment as shown in Fig. 4 but here coacervate samples are incubated for 4 *h* at 48°C. Due to longer incubation timings, here the composition is perturbed to the same extent as shown in Fig. 4A. All the measurements are done in triplicates and mean WXYZ product formation is plotted as fraction w.r.t. each node along with standard deviation.

REFERENCES

[1] Hayden, E. J. & Lehman, N. Self-Assembly of a Group I Intron from Inactive Oligonucleotide Fragments. *Chem. Biol.* **13**, 909–918 (2006).

[2] Hayden, E. J., von Kiedrowski, G. & Lehman, N. Systems chemistry on ribozyme self-construction: evidence for anabolic autocatalysis in a recombination network. *Angew. Chem. Int. Ed.* **120**, 8552–8556 (2008).

[3] Vaidya, N. *et al.* Spontaneous network formation among cooperative RNA replicators. *Nature* **491**, 72–77 (2012).

[4] Ameta, S. *et al.* Darwinian properties and their trade-offs in autocatalytic RNA reaction networks. *Nat. Commun.* **12** (2021).

[5] von Kiedrowski, G. Minimal Replicator Theory I: Parabolic Versus Exponential Growth. *Bioorg. Chem. Front.* 113–146 (1993).

[6] Ameta, S., Matsubara, Y. J., Chakraborty, N., Krishna, S. & Thutupalli, S. Self-Reproduction and Darwinian Evolution in Autocatalytic Chemical Reaction Systems. *Life* **11**, 308 (2021a).

[7] Poudyal, R. R. *et al.* Template-directed RNA polymerization and enhanced ribozyme catalysis inside membraneless compartments formed by coacervates. *Nat. Commun.* **10**, 1–13 (2019).

[8] Szer, W. Effect of di- and polyamines on the thermal transition of synthetic polyribonucleotides. *Biochem. Biophys. Res. Commun.* **22**, 559–564 (1966).

[9] Kamimura, A., Matsubara, Y. J., Kaneko, K. & Takeuchi, N. Horizontal transfer between loose compartments stabilizes replication of fragmented ribozymes. *PLoS Comput. Biol.* **15**, 1–15 (2019).

[10] Yeates, J. A., Hilbe, C., Zwick, M., Nowak, M. A. & Lehman, N. Dynamics of prebiotic RNA reproduction illuminated by chemical game theory. *Proc. Natl. Acad. Sci. U.S.A* 5030–5035 (2016).
